# A Comprehensive Assessment of Physiological and Neural Changes Underlying Cognitive Performance after Heat Stress: A Randomized Clinical Trial

**DOI:** 10.1021/envhealth.5c00818

**Published:** 2026-06-25

**Authors:** Lu Zhou, Zhang Shi, Ye Yao, Xihao Du, Xinlei Zhu, Yixiang Zhu, Shuai Xu, Jiang Lin, Renjie Chen, He Wang, Haidong Kan

**Affiliations:** † School of Public Health, Key Lab of Public Health Safety of the Ministry of Education, NHC Key Lab of Health 8 Technology Assessment, 12478 Fudan University, Shanghai 200032, China; ‡ Shanghai Key Laboratory of Meteorology and Health, Shanghai 200030, China; § Department of Radiology, Zhongshan Hospital, 92323 Fudan University, Shanghai 200032, China; ∥ Shanghai Institute of Infectious Disease and Biosecurity, School of Public Health, 12478 Fudan University, Shanghai 200032, China; ⊥ Department of Epidemiology and Biostatistics, School of Public Health, Shanghai Jiao Tong University School of Medicine, Shanghai 200030, China; # Human Phenome Institute, Institute of Science and Technology for Brain-Inspired Intelligence, Key Laboratory of Computational Neuroscience and Brain-Inspired Intelligence, 12478 Fudan University, Shanghai 200433, China

**Keywords:** heat stress, randomized, crossover, controlled trial, cognitive function, neurobehavioral test, functional magnetic resonance imaging, transcriptome

## Abstract

Heat stress threatens neurological health as a risk factor for cognitive decline. However, deeper understanding of the psychological, cognitive, and neural changes induced by heat stress, with stronger causal inference and biological evidence, is needed. A randomized, crossover, controlled trial was conducted among 30 healthy adults (15 males, 15 females; mean age 21.5 ± 1.5 years) during October–November 2022. Participants were randomly divided into two groups and alternately underwent 2-h exposures to high temperature (32 °C) and moderate temperature (22 °C) at a laboratory in Shanghai, China. Psychological distress was evaluated using a simplified DASS-21 scale. Cognitive function was assessed through neurobehavioral tests (Schulte grid and Stroop tests), with neural activity examined using functional magnetic resonance imaging (fMRI) to assess blood oxygen level-dependent signals. Blood samples were collected for plasma exosome transcriptomics and targeted biomarker analyses. Acute heat exposure significantly increased psychological distress by 27.3%, decreased processing speed by 13.6%, and impaired response inhibition by 3.4%. Heat stress reduced neural activity and synchronization in the precentral gyrus, inferior frontal gyrus, and rostral hippocampus. Functional connectivity was also attenuated in these regions. These alterations might be driven by type I interferon-dominated inflammation, excitotoxicity, and oxidative stress. Conversely, neural regulatory responses were observed, including activation of the lateral occipital cortex and elevated levels of neurotrophic factors. This trial demonstrates the relationship between acute heat exposure and diminished cognitive function. These findings underscore the need for public health strategies to maintain indoor temperatures within a comfortable range and promote active cooling to protect cognitive health.

## Graphical abstract

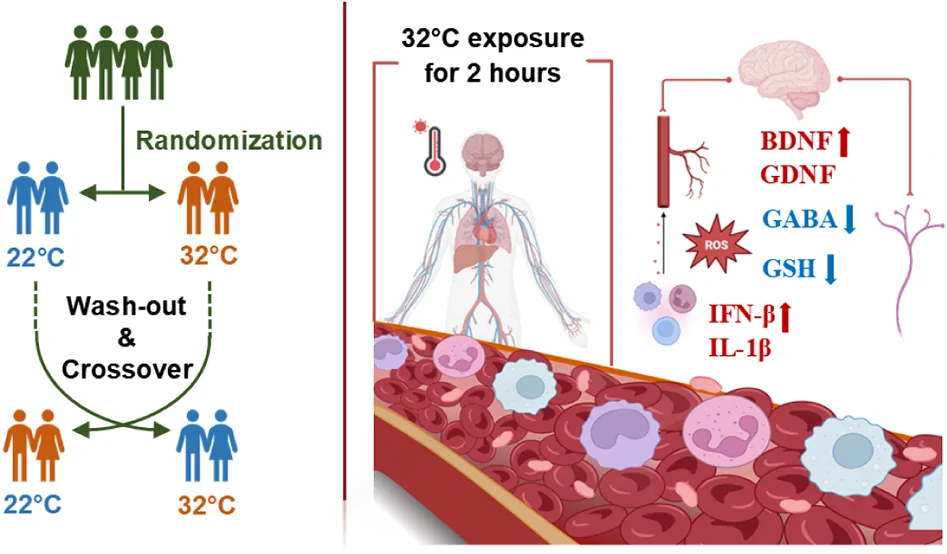

## 1 Introduction

Amid accelerating climate change, rising temperatures have elevated heat stress to a globally significant public health issue.^1,2^ Beyond physical health effects, heat stress may impair the integrity of higher-order cognitive functions, the multidimensional mental abilities and processes, including attention, memory, learning, problem-solving, and decision-making. Cognitive impairment is associated with worse health-related quality of life and higher morbidity and mortality risks of neurological diseases such as dementia.^3,4^ Additionally, the related medical, social, and economic burdens are projected to worsen over time.^5^ Due to rapid population aging, cognitive impairment imposes a growing challenge to public health globally.^4–8^ For instance, the number of individuals with dementia is projected to increase from 57.4 million in 2019 to 152.8 million in 2050 worldwide.^7^ While the projected increase in long-term neurological risks underscores a critical public health challenge, addressing this complexity requires a foundational understanding of the immediate responses to heat stress. Consequently, a characterization of acute cognitive and neural responses represents an essential first step toward a more comprehensive assessment of heat-related effects.

Beyond traditional risk factors,^4,7^ emerging evidence highlights hot temperature as a significant risk factor for cognitive decline and relevant diseases.^9^ More specifically, previous investigations indicate that heat stress is associated with adverse shifts in emotional states, such as anxiety and stress.^10,11^ Heat stress may also selectively impair executive functions, including attention control and response inhibition, often assessed using tasks such as the Stroop test.^12,13^ Concurrently, neuroimaging investigations have revealed the neural underpinnings of these deficits, showing that heat exposure may alter neural activity patterns in critical brain regions.^14–16^ While short-term heat exposure has been linked to transient reductions in cognitive performance,^17–21^ the immediate cognitive and neural mechanisms underlying these acute impairments require further investigation.

Existing evidence on the relationship between heat stress and cognitive function is mostly derived from observational investigations^17–21^ and is often confounded by concomitant exposures to air pollution, noise, and green space, which are difficult to account for or eliminate in the real world.^18^ While several interventional studies have attempted to assess the cognitive effects of heat exposure,^14–16,22,23^ they often employed an artificial exposure mode and level, such as simulating extreme heat through thermal laboratory coveralls^14–16,22^ or hot water immersion,^23^ which could not replicate the real-world heat stress conditions. Furthermore, the application of advanced neuroimaging tools like functional magnetic resonance imaging (fMRI) to capture neural responses to heat in humans has been limited due to high requirements of equipment and technologists.^14–16^ Notably, fMRI, a noninvasive mainstay of neuroimaging in cognitive neuroscience, enables holistic monitoring of brain structure and function.^24^ With high spatiotemporal resolution via blood oxygenation-level-dependent (BOLD) signal detection, fMRI could capture alterations of neural activity and early subclinical neural disruptions preceding the manifestation of cognitive impairment.

From a theoretical perspective, adverse neural effects of heat stress are mediated by a complex network of biological mechanisms, primarily involving homeostasis disruption and subsequent pathological events.^7,25,26^ It has been hypothesized in the broader literature that repeated or prolonged induction of these pathological events could accumulate over time, resulting in neurological damage that accelerates the progression of cognitive impairment,^1,2,9^ though this hypothesis remains speculative and requires longitudinal investigation. At the molecular level, animal experiments reported that even moderate heat stress within the range of compensatory thermoregulation can elicit oxidative stress through mitochondrial dysfunction and the generation of reactive oxygen species.^27–29^ Concurrently, heat stress could trigger neuroinflammatory responses, including microglial activation and the release of pro-inflammatory cytokines.^29,30^ However, existing mechanistic studies have yet to fully establish the direct link between acute heat exposure and cognitive dysfunction in humans or to elucidate the immediate adaptive physiological responses and the underlying regulatory mechanisms during short-term exposure episodes.

The present study focuses on the foundational characterization of acute cognitive and neural responses during single-exposure episodes. To address the current knowledge gaps regarding acute heat-induced cognitive impairment, our study offers several key advancements. First, we employ a randomized, crossover design in a controlled human exposure setting, providing a robust framework that overcomes the confounding inherent in observational studies. Second, our exposure protocol is designed to mimic real-world heat stress conditions, enhancing the ecological validity of our findings beyond artificial laboratory simulations. Third, we pioneer the integrated use of fMRI to identify heat-susceptible brain regions and characterize acute alterations in multilevel functional metrics. Finally, to elucidate potential immediate molecular mechanisms, we perform plasma exosome transcriptome analysis to evaluate acute heat-induced gene expression changes, with subsequent validation through targeted serum biomarker assessments.

## 2 Methods

### 2.1 Study Design and Participants

This randomized, controlled, crossover trial was conducted from October to November 2022 in Shanghai, China. We initially recruited 36 right-handed students from the Fenglin campus of Fudan University. All participants were healthy young adults with a body mass index (BMI) between 18.5 kg/m^2^ and 28.0 kg/m^2^, who did not smoke, drink, take drugs, or take dietary supplements during the study period. We excluded those with a history of allergic, cardiovascular, and respiratory diseases or neurological disorders at enrollment. Eligible participants were randomly assigned to two groups (1:1) using a computer-generated randomization sequence and then underwent two separate exposure sessions (high temperature: 32 °C; moderate temperature: 22 °C) in turn, i.e., one group experienced the high-temperature session first, followed by the moderate-temperature session, while the other group experienced the two sessions in the opposite order. This counterbalanced crossover design was specifically employed to minimize potential period and sequence effects and to account for stable between-subject characteristics. However, it is recognized that within-subject state-dependent factors, such as subtle variations in hydration or fatigue, might still introduce residual variability across sessions. Additionally, given that this trial was conducted during mild autumn months in Shanghai with participants who were primarily indoor-dwelling students, we assumed minimal heat acclimatization, though this was not formally measured or verified. Outcome assessors were blinded to the exposure assignment to minimize potential bias. A wash-out period of at least 2 weeks was maintained between sessions to eliminate any potential carry-over effects. The sample size was calculated a priori using PASS 15 software (NCSS, LLC, Kaysville, UT). The detailed procedure was described in the Supplementary Methods. Additionally, sample size determination was informed by previous studies.^31,32^ Overall, a total of 20 participants would provide sufficient statistical power to detect the effect.

The high temperature setting of 32 °C was intended to simulate real-world indoor summer heat exposure in non-air-conditioned environments (no solar radiation and wind), which could induce marked thermal discomfort and measurable physiological responses without compromising participant safety.^31,33,34^ The moderate temperature of 22 °C was selected as the control condition, as it falls within the thermoneutral zone for resting humans and is widely used as a neutral comparator in physiological research.^35^ In the controlled exposure setting, relative humidity was set at 50%, and indoor air pollutants were reduced using air purifiers. The 2-h exposure duration was informed by a previous study reporting detectable cognitive effects of 32 °C heat exposure within this time frame.^31^ During the exposure session, all participants were requested to remain seated and quiet in the chamber with their hands on the table. To ensure comparability, both exposure sessions were scheduled for a fixed time on the same day (i.e., 14:00 to 16:00). Similarly, we simultaneously performed cognitive examinations before exposure, during the period of exposure, and after exposure for each session. To control for the impact of diet, we provided the same meals and water for all participants on the day of the exposure session. Hydration was partially standardized by providing 200 mL of purified water during each 2-h exposure session, and participants were instructed to maintain their habitual hydration during the study period. However, objective measures of actual hydration status were not obtained.

Throughout each exposure session, participants were continuously accompanied by trained research staff inside of the exposure chamber. A physician was stationed outside the chamber to monitor the participant’s safety and provide immediate medical care. Prespecified termination criteria included participant request to withdraw, observed signs of heat-related distress (e.g., excessive sweating, confusion, nausea), or any adverse event requiring medical attention. No participant met these termination criteria during the study, and no adverse events were reported. The study protocol was approved by the Institutional Review Board at the School of Public Health, Fudan University (IRB#2022-04-0955) and registered at ClinicalTrials.gov (NCT05575752). Informed written consent was obtained from all participants at enrollment.

### 2.2 Exposure Measurement

Ambient temperature and relative humidity in the chamber were detected using the HOBO logger (Onset Computer Corporation, Pocasset, MA). We also monitored the real-time exposure to air pollutants throughout the exposure session. The concentrations of fine particles with a diameter less than 2.5 μm (PM_2.5_) were measured by microPEM (RTI International, USA); gaseous pollutants such as nitrogen dioxide (NO_2_) and ozone (O_3_) were measured by Personal Exposure Kit (PEK-lite A1, Sapiens Environmental Technology Co. Limited, Hong Kong, China). Additionally, the skin temperature of each participant was continuously monitored using a small button-type logger fastened to the palmar side of the nondominant wrist with hypoallergenic medical tape. This approach avoids potential confounding from invasive core temperature monitoring that may influence participant comfort or cognitive function assessment and serves as a direct physiological indicator of thermal strain.

### 2.3 Psychological Distress Measurement

A simplified version derived from the 21-item Depression Anxiety and Stress Scale (DASS-21) was used to assess psychological distress level. As shown in the Supplementary Methods, only 9 items suitable for assessing the impact of heat stress were included in this study. Participants were required to rate their current feelings on a 4-point Likert scale from 1 (not at all) to 4 (completely). A higher cumulative score indicated a higher level of psychological distress. The internal consistency of the 9-item adapted scale was good, with Cronbach’s α = 0.83 (95% CI: 0.76, 0.89) based on baseline assessments.

### 2.4 Cognitive Function Assessment

All participants were required to complete two cognitive tests using their mobile phones before exposure, after 1-h exposure, and after 2-h exposure in each session. Using their own devices ensured that each participant experienced an identical interface and settings across all tests. This approach minimized device-related variability by holding device characteristics (e.g., screen size and touch sensitivity) constant within participants, thereby enhancing the comparability between two exposure sessions. Additionally, all tests were web-based using standardized code, ensuring identical stimulus presentation and response registration across all devices. Detailed descriptions of assessments are provided in the Supporting Information.

In brief, the Schulte grid test evaluated visual attention and processing speed. Participants were presented with a 5 × 5 table with randomly arranged numbers from 1 to 25 and asked to tap the numbers in sequence on the screen as quickly as possible.^36^ Completion time reflected selective attention capacity and psychomotor speed. Two response time metrics were extracted: the shortest time to complete a task and the average time to complete the entire test. The Stroop color-word test assessed response inhibition and cognitive interference through congruent, incongruent, and neutral trials.^12,13^ Participants were instructed to indicate the color of the presented stimuli by pressing the corresponding on-screen button. The mean time of correct responses reflected the ability to resolve cognitive conflict and inhibit prepotent responses, and overall accuracy was calculated as the proportion of correct responses out of all trials. Additionally, the aforementioned measures were computed separately for each type of trial.

To ensure familiarity with the tasks, participants received detailed instructions and task requirements 3 days before each exposure session and were requested to practice twice daily. Through repeated practice, all participants acquired the requisite proficiency and mastery of the tasks.

### 2.5 fMRI Scanning Procedure and Imaging Data

Immediately following exposure, a randomly preselected subset of participants (determined by the initial randomization to avoid self-selection bias) underwent fMRI scanning at the Radiology Department of Zhongshan Hospital affiliated with Fudan University, located within 100 m of the exposure chamber. To minimize potential confounding effects from outdoor environmental conditions, participants were transported by a thermostatic vehicle to avoid uncontrolled exposure during transit. fMRI scanning was not performed for all participants because of constraints related to machine availability, technician capacity, and operating time. The detailed fMRI scanning procedures are provided in the Supporting Information. Briefly, an axial gradient-echo planar imaging (EPI) was acquired on a Philips Ingenia CX 3.0T MRI scanner. Participants were instructed to remain relaxed with their eyes open without the need to focus on any task. Dual-echo gradient-echo EPI was acquired to enhance the sensitivity.

For functional imaging data analysis, the detailed information was provided in the Supplementary Methods. We followed a classical data analysis framework using SPM12 (Statistical Parametric Mapping) with the DPABI toolbox.^37^ Three fMRI metrics were extracted to reflect distinct aspects of neural activity and connectivity, including fractional amplitude of low-frequency fluctuation (fALFF), degree of centrality (DC), and regional homogeneity (ReHo). An overview of these metrics is provided in Figure S1. In brief, fALFF reflects the local spontaneous neural activity intensity, DC quantifies a region’s centrality and functional connectivity strength within the whole-brain network, and ReHo measures local synchronization of neural activity among neighboring voxels. Together, these metrics characterize distinct dimensions of neural function.

### 2.6 Blood Collection and Sample Preparation

We collected intravenous blood from each participant before and after each exposure session. For transcriptomics analysis, blood samples were collected using EDTA anticoagulation tubes (BD Vacutainer EDTA Tube), while serum samples intended for ELISA experiments were obtained using procoagulation tubes (BD Vacutainer SST Serum Separation Tube). All specimens were immediately placed on ice after collection to prevent sample deterioration. The samples were subsequently stored at −80 °C until analysis.

### 2.7 Transcriptome Analysis

We only used after-exposure blood samples for the transcriptome analysis because of the limited budget. The detailed procedures of exosome isolation and identification, RNA extraction and sequencing, and data processing are articulated in the Supporting Information. Exosomes were isolated from plasma by using the exoEasy Maxi Kit (Qiagen, Hilden, Germany) and identified. Total RNA was extracted from exosomes, and strand-specific RNA sequencing libraries were constructed with the SMARTer Stranded Total RNA Kit (Clontech, Palo Alto, CA). The resulting libraries were sequenced on an Illumina platform (San Diego, CA). After quality control and data processing, gene expression levels from RNA sequencing were presented as mRNA counts and converted to transcripts per kilobase of million (TPM).

### 2.8 Targeted Biomarkers

ELISA kits (Fine Biotech) were used to assess the changes in targeted biomarkers, including brain-derived neurotrophic factor (BDNF), glial cell line-derived neurotrophic factor (GDNF), gamma-aminobutyric acid (GABA), interleukin-1β (IL-1β), and interferon-β (IFN-β). Additionally, glutathione (GSH) levels were assessed using the Glutathione Assay Kit (MLBio). To avoid interplate variability, all serum samples from the same participant, including before- and after-exposure samples in each exposure session, were analyzed sequentially on the same plate.

### 2.9 Statistical Analyses

We explored the effects of heat exposure on multiple health outcomes related to cognitive function. To account for baseline differences, percentage changes from pre- to postexposure periods (including 1-h and 2-h) were calculated for measures of psychological distress and cognitive function. Linear mixed-effects (LME) models were applied to assess the difference of changes between the two exposure sessions, with a fixed-effect binary indicator of exposure and a random-effect intercept for each participant. Additionally, age, sex, and BMI were adjusted for as fixed-effect covariates in the models. The same statistical approach was applied for the analysis of targeted biomarkers. Effect estimates were reported as percentage differences with 95% confidence intervals (CIs) associated with the heat exposure. Furthermore, exploratory analyses were conducted by adding a sex × intervention interaction term to the models to evaluate potential sex differences in heat responses. To visualize individual-level response patterns, within-subject changes in several key psychological and cognitive outcomes were plotted for each participant across exposure sessions.

For fMRI metrics, LME models were conducted without baseline adjustment, with effect estimates expressed as percentage differences with 95% CIs comparing high-temperature to moderate-temperature sessions. False discovery rate (FDR) correction with the Benjamini–Hochberg method was applied to control for the inflated type I error rates, and a two-sided FDR value of <0.1 was considered significant.^38^ Brain regions sensitive to heat exposure were identified and defined based on a stringent dual criterion: the region must exhibit a significant alteration in at least one fMRI metric after heat exposure, and the alteration must be significantly correlated with the corresponding change in at least one measure of cognitive function. Then, these identified regions were subsequently annotated using the brain connectome atlas (Table S1).

Transcriptome analysis involved orthogonal partial least-squares discriminant analysis (OPLS-DA) followed by LME models to identify differentially expressed genes after heat exposure, with variable importance in the projection (VIP) more than 1, the absolute value of fold change more than 1.5, and FDR value less than 0.1 to provide more information. Pathway enrichment analysis was conducted on differentially expressed genes associated with cognitive and neurological functions using Gene Ontology (GO).

## 3 Results

### 3.1 Descriptive Statistics

We conducted a randomized, controlled, crossover trial from October to November 2022 in Shanghai, China. Figure S2 illustrates the flowchart of participation in enrollment. A total of 30 healthy young adults (15 males and 15 females) completed two exposure sessions and cognitive tests. The six participants who did not complete the study were excluded for the following reasons: smoking (*n* = 2, did not meet inclusion criteria) and scheduling conflicts or personal reasons prior to first exposure (*n* = 4). No participant withdrew or was excluded during or after any exposure session. All participants reported no medication use, dietary supplement, active or passive smoking, or alcohol consumption during the study period. As shown in Table 1, the average age of the participants was 21.5 [standard deviation (SD) = 1.5] years, and the average BMI was 21.7 (SD = 1.9) kg/m^2^. Of the 30 participants, 11 males and 11 females completed MRI tests after both exposure sessions. The baseline demographic characteristics of the 22 participants were comparable to those of the 30 participants (Table 1).

**Table 1:** Demographic Characteristics of the Participants with and without MRI Test^a^

Variable	All participants	With MRI test	Without MRI test	Between-groups *P*-values
N	30	22	8	-
Sex (*n*)				
Male	15	11	4	-
Female	15	11	4	-
Age, mean (SD), years	21.5 (1.5)	21.3 (1.6)	21.9 (1.4)	0.29
BMI, mean (SD), kg/m^2^	21.7 (1.9)	21.7 (2.0)	21.6 (2.0)	0.91

a MRI, magnetic resonance imaging; BMI, body mass index; and SD, standard deviation. The between-group *P*-values were assessed by *t*-test.

As shown in Table S2, during the exposure sessions, the average temperatures of the heat and control sessions were 32.1 °C (SD = 0.5) and 21.8 °C (SD = 0.3), respectively, while the corresponding relative humidity values were 51.1% (SD = 1.8) and 50.7% (SD = 1.4). Additionally, the average skin temperatures of the two sessions were 33.6 °C (SD = 0.6) and 29.5 °C (SD = 1.2), respectively. The mean concentrations of air pollutants (PM_2.5_, NO_2_, and O_3_) were controlled at low levels, without obvious differences between two exposure sessions. During the study period, ambient outdoor temperatures remained stable and were consistently close to the level of the control session, and the background exposure conditions were comparable between the two exposure sessions (Table S3).

### 3.2 Heat Exposure and Cognitive Function

Prior to both exposure sessions, baseline measures of psychological distress and cognitive function of the participants were quite similar (Table 2). Table 2 shows the differences in cognitive function with heat exposure, while Table S4 provides descriptive statistics (mean ± SD) for changes from baseline to postexposure in all measured outcomes for both exposure sessions. By comparing the before-and-after difference between the two exposure sessions, we observed elevated psychological distress levels, slower responsiveness, and less accuracy associated with heat exposure. Specifically, the average psychological distress score increased 20.8% (95% CI: 8.7%, 32.9%) and 27.3% (95% CI: 8.7%, 45.9%) after 1-h and 2-h heat exposure. For the Schulte grid test, the average time to complete a task slightly increased 5.1% (95% CI: 0.7%, 9.5%) and 3.3% (95% CI: −1.7%, 8.3%) after 1-h and 2-h heat exposure. Accordingly, there were increases of 9.4% (95% CI: 3.2%, 15.6%) and 13.6% (95% CI: 6.2%, 20.9%) in the shortest time to complete a task. For the Stroop test, we observed increases of 2.9% (95% CI: 0.8%, 4.9%), 2.5% (95% CI: 0.0%, 4.9%), 3.4% (95% CI: 1.1%, 5.7%), and 2.0% (95% CI: −0.7%, 4.7%) in response time for all, congruent, incongruent, and neutral trials after 2-h heat exposure, respectively. Overall, the performance of incongruent trials was most significantly affected by heat exposure. In addition, we observed a negative but nonsignificant association between heat exposure and the accuracy of response, with decreases of 0.5% and 0.3% in accuracy after 1-h and 2-h exposure, respectively.

**Table 2:** Baseline Statistics and Percentage Changes in Psychological Distress Score and Neurobehavioral Indicators Comparing the Heat-Exposure to the Control-Exposure Sessions (*n* = 30)^a^

	Baseline levels (SD)		1-h exposure		2-h exposure	
Variable	Heat exposure	Control	Δ% (95% CIs)	*P*-value	Δ% (95% CIs)	*P*-value
**Psychological distress score**	9.0 (3.5)	10.1 (4.2)	20.8 (8.7, 32.9)	0.002	27.3 (8.7, 45.9)	0.006
Schulte grid test						
Response time (s)						
Average time	22.10 (4.68)	21.32 (4.10)	5.1 (0.7, 9.5)	0.027	3.3 (−1.7, 8.3)	0.196
Shortest time	19.11 (4.75)	18.79 (4.14)	9.4 (3.2, 15.6)	0.005	13.6 (6.2, 20.9)	<0.001
Stroop test						
Response time (ms)						
All	803.92 (76.19)	823.69 (71.96)	2.0 (−0.3, 4.2)	0.105	2.9 (0.8, 4.9)	0.008
Congruent	775.43 (75.19)	794.55 (69.51)	1.4 (−0.9, 3.8)	0.239	2.5 (0.0, 4.9)	0.049
Incongruent	831.12 (80.34)	851.84 (80.13)	2.7 (0.3, 5.1)	0.034	3.4 (1.1, 5.7)	0.005
Neutral	780.88 (82.32)	799.25 (68.54)	0.7 (−2.5, 3.8)	0.677	2.0 (−0.7, 4.7)	0.155
Accuracy (%)						
All	96.2 (2.2)	95.5 (2.5)	–0.5 (−1.5, 0.6)	0.403	–0.3 (−1.3, 0.7)	0.568
Congruent	98.0 (2.0)	97.8 (2.0)	0.9 (−0.4, 2.3)	0.188	–0.2 (−1.5, 1.1)	0.757
Incongruent	94.7 (3.7)	93.6 (4.3)	–1.4 (−3.3, 0.5)	0.159	–0.2 (−2.1, 1.7)	0.816
Neutral	97.0 (2.6)	96.2 (3.3)	–0.6 (−2.8, 1.6)	0.594	–0.7 (−2.9, 1.6)	0.572

a SD, standard deviation; CI, confidence interval. Δ% (95% CIs) represents the percentage changes and their corresponding 95% confidence intervals.

Notably, no significant effects of sex or sex × intervention interactions were observed for any psychological or cognitive outcome (Table S5), indicating that cognitive responses to heat stress did not differ significantly between males and females in this study. Additionally, while group-level analyses revealed significant heat-induced cognitive impairment, individual response patterns exhibited considerable heterogeneity, with the magnitude of impairment varying substantially across participants (Figure S3).

### 3.3 The Profile of fMRI Metrics and Differential Brain Regions

The fMRI profile associated with heat exposure suggested consistent changes across the left and right brain hemispheres (Figure S4). The corresponding anatomical and modified cyto-architectonic descriptions for brain regions are presented in Table S1. We finally identified eight (out of 123) brain regions that responded differently to heat and moderate temperatures. These regions were scattered in the inferior frontal gyrus, precentral gyrus, lateral occipital cortex, and hippocampus (Figure 1). Generally, there were decreased fMRI metrics (fALFF, DC, and ReHo) derived from most brain regions in relation to heat exposure except for the lateral occipital cortex. For instance, fALFF decreased significantly in the precentral gyrus, ranging from 16.0% to 60.9% across different regions. Similarly, DC decreased significantly in the inferior frontal gyrus, ranging from 28.4% to 79.6%, and ReHo was 18.8% (95% CI: 8.8%, 28.7%) lower in the left rostral hippocampus. For the lateral occipital cortex, we observed elevated fALFF and DC, but neither reached statistical significance. Additionally, ReHo was 53.6% (95% CI: 18.8%, 88.3%) and 32.7% (95% CI: 12.1%, 53.3%) higher after heat exposure in the right middle occipital gyrus and area V5/MT+, respectively (Table S6).

**Figure 1:**
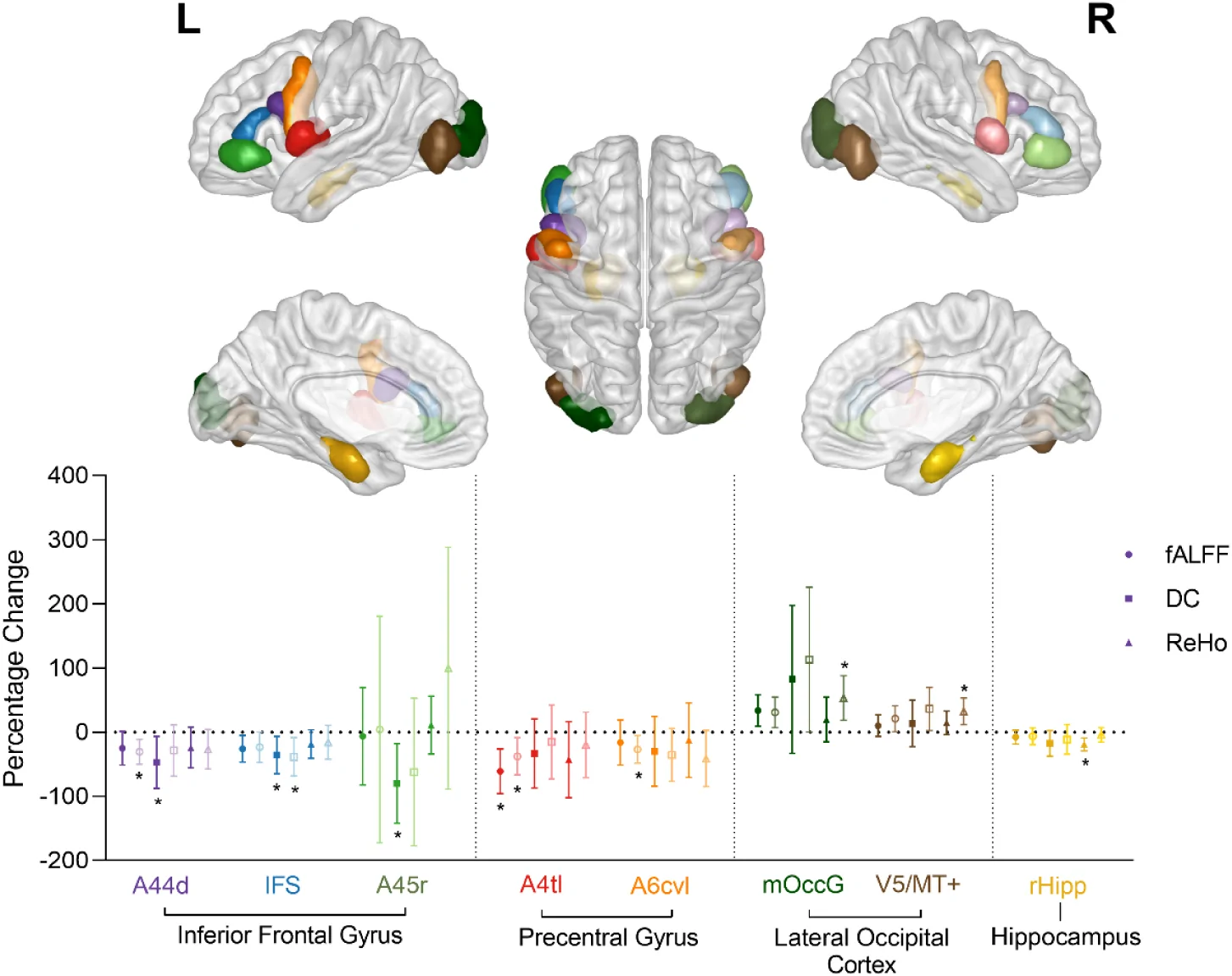
Identification of the differential brain regions and percentage changes in fMRI metrics comparing heat-exposure to control-exposure sessions, with the same colors annotating spatial locations and corresponding results, and color shades representing left and right brain hemispheres. * *FDR-value* < 0.1.

### 3.4 Untargeted Transcriptomic Profiling of Plasma Exosomes

The isolated exosomes were examined and validated, showing typical cup-shaped vesicles and a particle size distribution consistent with exosomal characteristics (Figure S5). After data curation and annotation, we finally included 12358 plasma exosome mRNAs in the analysis. OPLS-DA analysis suggested a distinct separation between heat and control exposure sessions (Figure S6). As shown in Figure S7, a total of 507 mRNAs were significantly associated with heat exposure, with 311 increasing and 196 decreasing after heat exposure compared to the control exposure. As shown in Figure 2A, pathway enrichment analysis identified significant alterations in neurological pathways following an acute heat exposure. The dysregulated key pathways included the following major categories: cellular stress response, inflammatory response (e.g., type I interferon signaling), glial cell activation, synaptic signaling, neurodevelopment, and oxidative stress damage.

**Figure 2:**
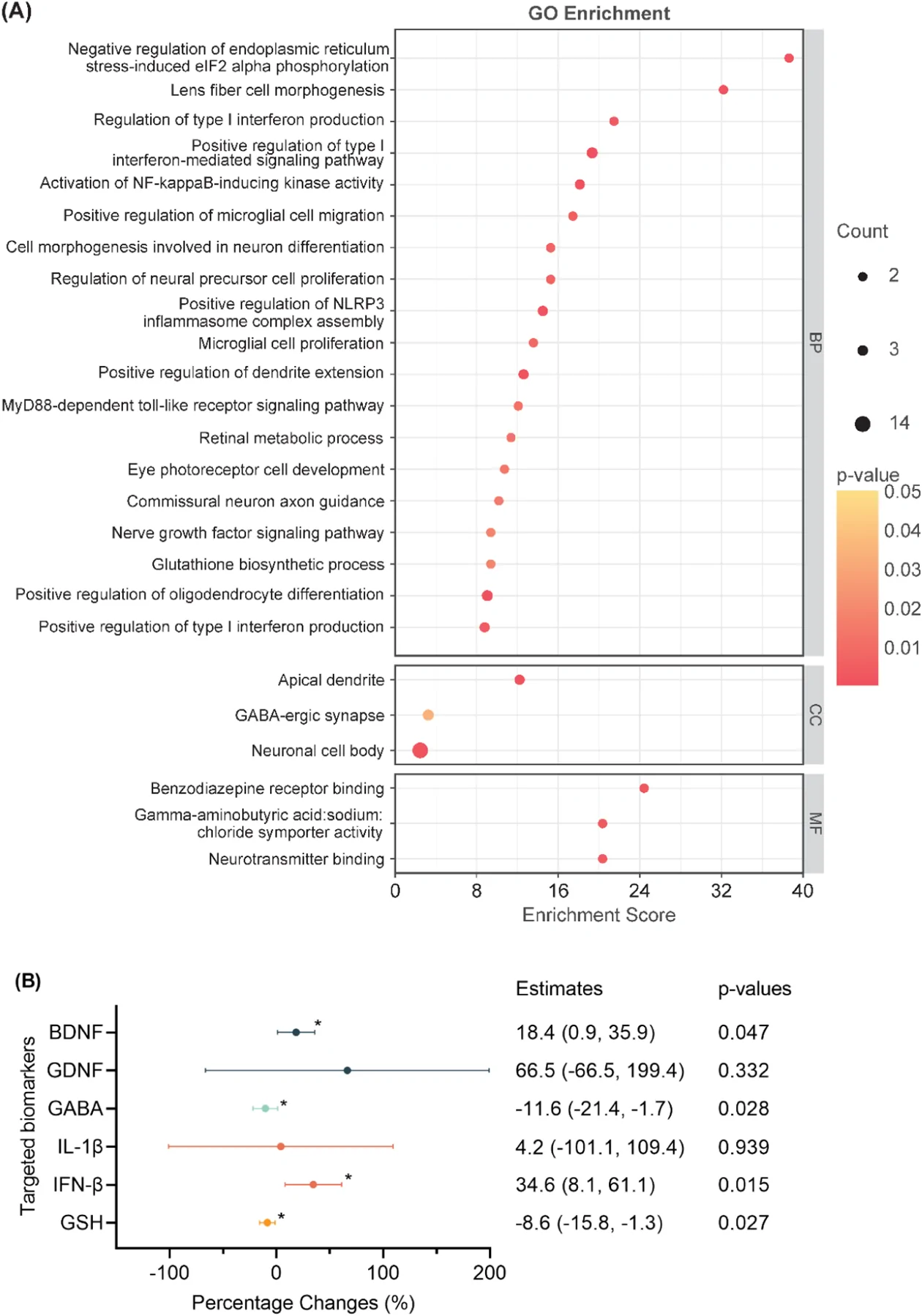
Integrated multiomics analysis of blood biomarkers after heat exposure. (A) Gene Ontology enrichment analysis of the plasma exosome transcriptome; (B) Percentage changes in targeted serum biomarkers comparing the heat-exposure to the control-exposure sessions.

### 3.5 Heat Exposure and Targeted Biomarkers

Compared to the control exposure, acute heat exposure significantly altered peripheral blood levels of targeted biomarkers (Figure 2B). Neurotrophic factors were elevated, with BDNF and GDNF increasing 18.4% (*p* = 0.047) and 66.5% (*p* = 0.332). Inflammatory biomarkers IL-1β and IFN-β exhibited increases of 4.2% (*p* = 0.939) and 34.6% (*p* = 0.015), respectively. Conversely, the neurotransmitter GABA decreased 11.6% (*p* = 0.028), alongside a reduction of 8.6% in the antioxidant marker GSH (*p* = 0.027).

## 4 Discussion

This randomized, crossover, controlled trial systematically explored the effect of heat stress on cognitive function and its underlying mechanisms through comprehensive assessment. Our results revealed significant neurobehavioral impairments in selective attention, inhibitory control, and processing speed under a thermally uncomfortable environment. Neuroimaging data provided empirical evidence of neurobehavioral responses, revealing that the inferior frontal gyrus, precentral gyrus, lateral occipital cortex, and hippocampus might be the main brain regions sensitive to heat stress. Furthermore, mechanistic investigations suggested that perturbations in neurotransmitter, oxidative stress, and inflammatory responses may contribute to the detrimental health effects of heat stress on cognitive function. Notably, potential neural adaptive responses were observed, suggesting a compensatory mechanism that may partially counteract the heat-induced neuronal impairment. These findings enhance the understanding of the neural effects and mechanisms linking heat stress and acute cognitive impairment in healthy young adults under controlled conditions, offering insights into the context of global warming.

Consistent with previous studies,^12,39^ the present trial found that heat exposure could impair cognitive function, represented by diminished selective attention, reduced inhibitory control, and slower processing speed. Our study reinforced the evidence by the randomized crossover design and careful control of major environmental factors, such as temperature, humidity, and air pollutants. Furthermore, we observed deteriorating response inhibition capacity and reduced processing speed with prolonged heat exposure, which indicated that heat acclimation would not occur within such a short time or thermal regulation would not sufficiently mitigate these cognitive impairments.^40^ However, several studies suggested that acute heat exposure would not alter simple task performance (orienting and alerting),^14^ decision-making performance,^41^ or attention.^42^ Methodological discrepancies across different studies have limited the ability to draw consistent conclusions on whether acute heat exposure has an adverse cognitive effect. The heterogeneity in the findings of prior randomized controlled trials might originate from the magnitude and duration of heat exposure, the complexity and duration of cognitive tasks, the approach of exposure (whole-body or local), the exposure pathway (through thermal laboratory coveralls, immersion in water, or air), the characteristics of the participants, and the time framework of the trials (seasonality). Nevertheless, we believe that our findings are more reliable because of the simultaneous consideration of the following aspects. First, we adopted a randomized controlled crossover study design and took full account of baseline cognitive performance, which facilitates accurate assessment of the heat effect. Second, we chose an appropriate exposure mode and level to simulate heat exposure typically encountered in indoor environments, thereby enhancing the ecological validity of our experimental findings in daily life. Third, we conducted the trial among healthy college students to minimize the potential confounding effects of socioeconomic characteristics, adverse behavioral factors, and medication/supplements. Finally, this trial was conducted within a short time frame, effectively controlling for the impacts of great variations in environmental conditions and all season-related unmeasured factors.

Notably, no significant sex differences in heat-induced cognitive impairment were observed despite established sex differences in thermoregulatory physiology. Females typically exhibit lower sweat rates, greater reliance on cutaneous vasodilation, and different autonomic and cerebrovascular responses to heat stress compared with males.^43,44^ The absence of sex-differentiated cognitive effects in our study may indicate that these physiological differences do not translate into differential cognitive vulnerability under the current moderate heat exposure in young healthy adults. Alternatively, our sample size may have been insufficient to detect small-to-medium interaction effects. More extreme thermal challenges or populations with compromised thermoregulatory capacity may be required to reveal sex differences in heat-related cognitive impairments.

Our investigation has unveiled intriguing alterations in neural activity across brain regions (including the inferior frontal gyrus, precentral gyrus, and hippocampus) after heat exposure, characterized by reduced fMRI indices. Notably, these decrements may signify a compromised neurovascular coupling in response to heat, which reflects a constriction of blood vessels and reduced oxygen supply,^45–47^ subsequently impairing cognitive function. Previous fMRI studies reported inconsistent evidence on activation of brain regions after heat exposure, which might derive from the differences in types of neurobehavioral tests, timing of the fMRI test (during or after heat exposure), exposure level, and characteristics of study participants.^14–16^ Previous studies have revealed some brain regions potentially involved in the response to heat exposure, which include the dorsolateral prefrontal cortex,^14,16^ frontal lobe,^14,15^ temporal lobe,^14,15^ occipital lobe,^14^ and parietal lobe.^14–16^ In alignment with prior research, our findings suggested the involvement of several brain regions in the frontal lobe in higher-order cognitive processes like executive functions, covering a group of skills (e.g., inhibitory control, attentional regulation, and working memory) integral to the cognitive tasks employed in this study.^48,49^ The hippocampus, a brain region pivotal to memory,^50^ might also participate in the regulation of working memory function.^51^ Prior studies have suggested that functional hippocampus-frontal lobe interactions play major roles during working memory tasks,^52^ and the working memory maintenance relies on the hippocampal-cortical oscillatory synchronization observed in encephalography.^51^ Overall, the heat-induced alterations in neural activity within the frontal area and hippocampus might be pivotal factors contributing to cognitive dysfunction.

The observed cognitive impairment after heat exposure may be mechanistically linked to the significant alterations in circulating biomarkers.^45,53^ Combining untargeted transcriptomic and targeted biomarker analyses, our findings revealed neurotransmitter signaling alteration, oxidative stress, and inflammation after acute heat exposure. GABA, a key inhibitory neurotransmitter, maintains excitatory and inhibitory balance in the nervous system, protecting against excitotoxicity and supporting neuronal survival.^54^ The decrease in GABA level represents neurotransmitter imbalance, potentially impairing neural circuit efficiency and cognitive processing.^55^ Consistent with previous studies,^30,31,56,57^ we found elevated levels of oxidative stress and inflammation after heat exposure, which might cause neuronal damage. Notably, while existing evidence on heat-induced inflammation has focused on specific biomarkers (e.g., tumor necrosis factor-α and interleukins), few studies have addressed type I IFN-driven inflammatory responses. IFN-β plays a key role in immune modulation and microglial regulation, contributing to the mediation of neuroinflammatory responses.^58,59^ Its elevation following heat exposure is biologically plausible and supports its potential as a novel heat-induced biomarker indicative of acute neuroinflammation.

Beyond the detrimental effects of heat stress, we identified potential neural adaptive responses. For instance, fMRI findings revealed heightened synchronization of neural activity and enhanced connectivity in the lateral occipital cortex, which is a critical region involved in visual processing and attention.^60^ It has previously been shown to exhibit heightened sensitivity to thermal stress, as evidenced by electroencephalography signals in the occipital lobe.^61^ Our findings may reflect a reorganization of neural resources to core sensory and attentional processes in response to heat stress driven by the nonuniformity and preservability of neural resource allocation.^62^ Concurrently, elevated levels of neurotrophic factors were observed. BDNF is essential for neuronal survival, synaptic plasticity, and cognitive function and serves as a key biomarker for chronic cognitive dysfunction and neurodegenerative diseases.^63^ Although its role in our study remains speculative, increased BDNF levels after heat exposure may reflect a compensatory neuroprotective mechanism, attenuating neural damage and promoting adaptive processes.^64,65^ While the functional implications of these alterations for long-term cognitive health require further investigation, they collectively indicate a coordinated, albeit possibly incomplete, adaptive response to heat exposure involving both central and peripheral pathways.

Collectively, we propose that acute heat exposure impairs cognitive function primarily through oxidative stress, inflammation, and disruption of neurotransmitter homeostasis, leading to altered neural activity in heat-sensitive brain regions. Concurrently, compensatory neuroprotective mechanisms are activated to partially mitigate neural dysfunction and promote adaptive processes. These dual pathways of neural disruption and adaptation are shown in Figure 3.

**Figure 3:**
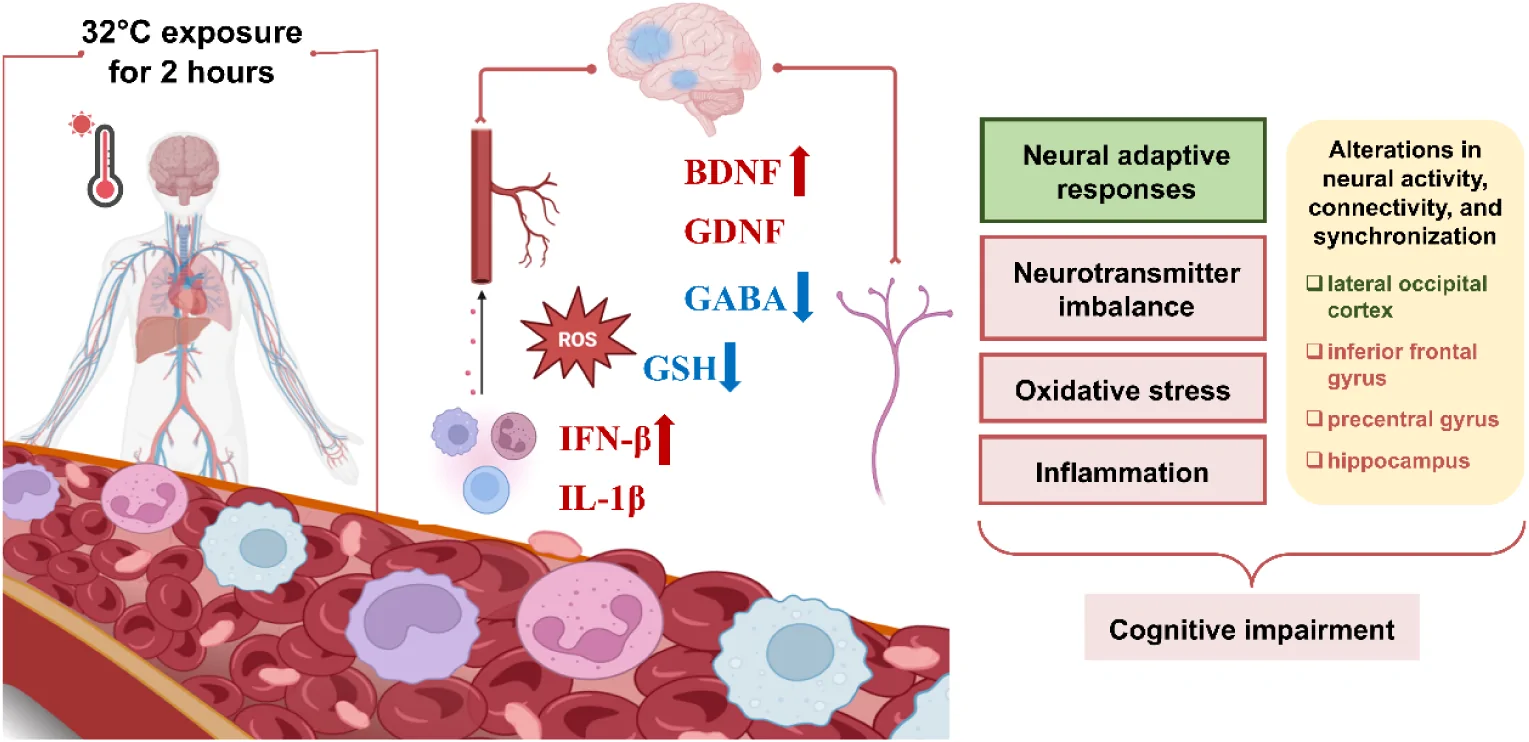
An illustrative overview of mechanisms underlying heat-induced cognitive dysfunction.

From a translational perspective, while our findings derive from controlled laboratory conditions in healthy young adults, they suggest that even this relatively resilient population exhibits measurable cognitive and neural alterations after acute heat exposure, underscoring the importance of active cooling in general populations. Although the long-term clinical significance of these acute alterations is not yet well-established, our findings support the recommendation that maintaining indoor temperatures below 26 °C through air conditioning or improved ventilation.^33^ Additionally, public health advisories should prioritize healthy buildings and accessible cooling strategies during heatwaves,^66,67^ including public cooling centers and targeted electricity subsidies, to mitigate acute neural impairment and enhance cognitive resilience in vulnerable subgroups.

Several limitations should be noted in this study. First, we only enrolled healthy young adults, which might limit the generalizability of the findings to more vulnerable populations such as older adults, children, individuals with comorbidities, or occupational groups. While sex is a critical biological variable known to influence thermoregulatory responses, this study was statistically underpowered to capture subtle sex-intervention interactions or sex-specific effects. Future studies with larger samples should clarify sex-related vulnerability to heat stress and examine potential mediation by sex hormones, menstrual cycle phase, or differences in thermoregulatory capacity. Second, given the ethical limitation, as done in previous trials,^19–21^ we set the intervention as 2-h heat exposure at 32 °C, which could adequately simulate indoor summer heat but could not capture the cumulative impact of prolonged heatwaves or more extreme outdoor exposure conditions involving other weather conditions (e.g., wind and solar radiation). Thus, our findings are most directly applicable to sedentary individuals in indoor settings and should not be overgeneralized to outdoor, active, or vulnerable populations without further investigation under more ecologically valid conditions. Third, skin temperature was monitored as a noninvasive surrogate for core body temperature to avoid confounding from invasive procedures. Future studies should incorporate minimally invasive core temperature monitoring to clarify its relationship with cognitive function. Fourth, our primary reliance on group-level averages may mask interindividual variability. While individual response patterns were presented, the small sample size precluded a statistical analysis of factors driving this heterogeneity, warranting further investigation on individual susceptibility to heat stress. Fifth, fMRI scanning and transcriptome analyses were performed after exposure, potentially limiting insight into real-time physiological dynamics and time-varying responses to heat stress. To mitigate intersession variability, all measurements were conducted at the same time of day, utilizing identical instruments and protocols, and participants were asked to ensure adherence to a stable lifestyle prior to each session. Sixth, fMRI assesses neural activity indirectly through BOLD signals, which reflect a hemodynamic surrogate rather than a direct measurement. This indirect approach, coupled with limited temporal resolution, may limit the interpretation of findings to hemodynamic correlates of neural function. Moreover, the lack of complementary techniques such as electroencephalography or diffusion tensor imaging may restrict a multidimensional understanding of functional network dynamics and structural integrity in the brain. Finally, it is so far insufficient to elucidate the mechanisms underlying the cognitive function impairment due to the difficulty in the collection of cerebrospinal fluid and brain specimens, and thus further validation and investigation are warranted in animal studies.

## 5 Conclusions

In summary, this randomized crossover trial demonstrated that acute heat exposure could significantly impair cognitive function. Furthermore, comprehensive integration of fMRI and mechanistic evidence linked neurobehavioral impairment to neuroimaging and peripheral physiological alterations, thereby strengthening the biological plausibility and credibility of the association between heat exposure and cognitive functions. Future studies should expand to broader populations and a wider range of cognitive processes to further elucidate the public health implications of heat exposure for neurological health.

## Supplementary Material


