# Supplementary material for: A Comprehensive Assessment of Physiological and Neural Changes Underlying Cognitive Performance after Heat Stress: A Randomized Clinical Trial
Source: Environ Health (Wash). 2026 Jun 25;4(10):2312–22. doi: 10.1021/envhealth.5c00818 (PMC13681429; doi:10.1021/envhealth.5c00818)
Supplement: Supplementary file 1 [file eh5c00818_si_001.pdf]

Supporting information

A Comprehensive Assessment of Physiological and Neural Changes Underlying Cognitive Performance after Heat Stress: A Randomized Clinical Trial

Lu Zhou[1,2†], Zhang Shi[3,†], Ye Yao[4,†], Xihao Du[1,5], Xinlei Zhu[1], Yixiang Zhu[1], Shuai Xu[6], Jiang Lin[3], Renjie Chen[1,*], He Wang[6,*], Haidong Kan[1,*]

[1]School of Public Health, Key Lab of Public Health Safety of the Ministry of Education, NHC Key Lab of Health 8 Technology Assessment, Fudan University, Shanghai, 200032, China;

[2]Shanghai Key Laboratory of Meteorology and Health, Shanghai, 200030, China;

[3]Department of Radiology, Zhongshan Hospital, Fudan University, Shanghai, 200032, China;

[4]Shanghai Institute of Infectious Disease and Biosecurity, School of Public Health, Fudan University, Shanghai, 200032, China;

[5]Department of Epidemiology and Biostatistics, School of Public Health, Shanghai Jiao Tong University School of Medicine, Shanghai 200030, China;

[6]Human Phenome Institute, Institute of Science and Technology for Brain-Inspired Intelligence, Key Laboratory of Computational Neuroscience and Brain-Inspired Intelligence, Fudan University, Shanghai, 200433, China.

† These authors contributed equally to this work.

***Correspondence to:**

Prof. Haidong Kan, Department of Environmental Health, School of Public Health, Fudan University, No.130 Dong-An Road, Shanghai, 200032, China. Tel/fax: +86 (21) 64046351. E-mail: kanh@fudan.edu.cn;

Prof. He Wang, Institute of Science and Technology for Brain-Inspired Intelligence, Fudan University, No.220 Handan Road, Shanghai, 200433, China. E-mail: hewang@fudan.edu.cn;

Prof. Renjie Chen, Department of Environmental Health, School of Public Health, Fudan University, No.220 Dong-An Road, Shanghai, 200032, China. E-mail:

chenrenjie@fudan.edu.cn.

Table of Contents

Supplementary Methods	Supplementary methods for sample size calculation, cognitive function assessment, fMRI scanning procedure and imaging data, and transcriptome
Table S1	Anatomical and modified cyto-architectonic descriptions for brain regions
Table S2	Descriptive statistics of exposure conditions during the exposure sessions
Table S3	Descriptive statistics of ambient exposure conditions during the three-day period preceding exposure
Table S4	Relative changes from baseline to post-exposure in psychological distress score and neurobehavioral indicators for the heat-exposure to the control-exposure sessions
Table S5	Sex effects and interaction effects on psychological distress score and neurobehavioral indicators
Table S6	Brain regions differentially involved in response to heat exposure and the corresponding percentage changes in fMRI metrics
Figure S1	Overview of functional MRI indices
Figure S2	Flowchart of participation in the present study
Figure S3	Individual response patterns across psychological and cognitive measures
Figure S4	Percentage changes in fMRI metrics comparing the heat-exposure to the control-exposure sessions
Figure S5	Exosome examination and validation
Figure S6	Score plot from the OPLS-DA analysis for the transcriptomic analysis

Figure S7 Volcano plot for the transcriptomic analysis

Supplementary Methods

1. Sample size calculation

The sample size was calculated a priori using PASS 15 software (NCSS, LLC, Kaysville, UT). The following formula was used for sample size calculation:

$$n = \frac{\left(z_{1-\alpha/2} + z_{1-\beta}\right)^2 \sigma^2}{2\delta^2}$$

n denotes the required sample size for each group;

α represents the type I error rate;

$1-\beta$ denotes the statistical power;

σ indicates the standard deviation of the difference in the primary outcome between the heat-exposure and control-exposure sessions, equivalent to the within-subject standard deviation of change;

δ refers to the mean difference in the primary outcome between the heat-exposure and control-exposure sessions.

In the PASS software, the procedure "Tests for the Difference Between Two Means in a 2×2 Cross-Over Design" was selected. The parameters were configured as follows:

$\alpha = 0.05$;

$1-\beta = 0.90$;

Mean difference under the null hypothesis (Mean Difference|H_0) was set to 0;

Mean difference under the alternative hypothesis (Mean Difference|H_1) was specified as Diff1;

Standard deviation of the difference between sessions was specified as S.

We calculated the sample size based on the primary outcome from Stroop test.

Diff1 was set at 10ms with a S of 10ms. The calculated sample size was 14. To account for potential dropouts and ensure feasibility, the sample size was inflated by 20%, resulting in a final requirement of 18 participants at enrollment.

2. Psychological distress measurement

Psychological distress was evaluated using a simplified instrument derived from the 21-item Depression Anxiety and Stress Scale (DASS-21). A subset of 9 items was selected to efficiently capture heat-related symptoms across the three domains of depression, anxiety, and stress. The included items were:

- *I found it hard to wind down*
- *I was aware of dryness of my mouth*
- *I experienced breathing difficulty (e.g., excessively rapid breathing, breathlessness in the absence of physical exertion)*
- *I tended to overreact to situations*
- *I felt trembling (e.g., in the hands)*
- *I found it difficult to work up the initiative to do things*
- *I found myself getting agitated*
- *I found it difficult to relax*
- *I felt that I was rather touchy*

The scale adopts a 4-point Likert scale ranging from 1 (not at all) to 4 (completely), and participants were required to select the option that best pertained to their current feelings. The total score from these 9 items was used as an indicator of overall

psychological distress, and a higher cumulative score indicated a higher level of psychological distress.

3. Cognitive function assessment

3.1 Schulte grid test

Schulte grid test was a classic tool used to measure the properties of attention, requiring visual scanning, flexible shifting, and motor speed. For each task, a 5×5 table with randomly arranged numbers from 1 to 25 was applied in this study, and participants were asked to identify and press the numbers in sequence on the screen. The time each participant took to complete the task was recorded as the measure of attention. Each complete test comprised of five tasks, each of which was followed by a 30-s resting time for preparation.

3.2 Schulte grid test

Stroop color-word test was applied to test cognitive inhibition. This task included three types of trials: congruent, incongruent, and neutral. For congruent trials, we used four color words (red, green, blue, and yellow) displayed in Chinese characters by the corresponding ink colors. For incongruent trials, we used the same color words and characters, but changed the ink colors to mismatched ones. For neutral trials, letters “XXXX” rather than Chinese characters were displayed in four ink colors. A complete 15-minute Stroop test consisted of five blocks of trials, each containing a total of 48 randomly displayed trials: 16 congruent, 24 incongruent, and 8 neutral trials. In each trial, the target stimuli (“+”) was first presented in the screen center for 500 ms. Then,

participants were instructed to indicate the color of the following presented stimuli as fast as possible within a response window of 1500 ms by pressing the button on the screen. An interval of 500 ms was set between trials.

4. fMRI scanning and imaging data

4.1 fMRI scanning procedure

An axial gradient-echo planar images (EPI) were acquired on a Philips Ingenia CX 3.0T MRI scanner. 240 time points were acquired during the 10 minutes resting phase wherein the subjects were instructed to keep their eyes open and to relax, without the need to focus on any particular task. Dual-echo gradient-echo echo-planar images (GE-EPI) were acquired to enhance sensitivity, using 8-channel SENSE head coil (SENSE factor 2, anterior-posterior direction, TE 30ms, flip angle 85°, 255 x 255 mm field of view, in-plane resolution = 3 mm x 3 mm, slice thickness = 2.9 mm, TR = 2000 ms; 40 descending axial slices, 240 time points per acquisition). Scans were inspected immediately after each acquisition, and if motion was detected, scans were repeated. In brief, head motion was assessed by calculating framewise displacement (FD) for each participant based on the realignment parameters. Our results indicated minimal head motion during scanning, and no participant exceeded the commonly accepted exclusion threshold of FD > 0.25 mm in any direction. Due to the slight head movements observed in the included participants, there was no repeated scan and movement could be effectively corrected in the data preprocessing. Axial slices parallel to the anterior commissure-posterior commissure plane acquired in ascending order. Acquisition was

performed for 400 volumes in one single session, of which the first six volumes were discarded for the analysis. Triggering of the stimulation by the scanner pulses was conducted using Presentation Software. Before functioning scanning, T1-weighted anatomical imaging was performed for each participant (with 1 mm isotropic resolution, 512 x 512 x 204 matrix, TR/TE 9.4/3.8 ms, shot interval 3 s, flip angle 12°, SENSE factor 2 was also acquired for each participant for image registration and to define anatomical regions for tractography). All procedures were conducted by experienced technologists from Zhongshan Hospital Affiliated to Fudan University.

4.1 Imaging data analysis

For functional imaging data analysis, the first five volumes of fMRI datasets were discarded, to allow for scanner stabilization. After that, the reconstructed and converted EPI images were realigned with the mean function image using SPM12 (Statistical Parametric Mapping) for movement correction. Then, slice time correction was carried out to minimize differences in acquisition times. The structural and reference images were co-registered, and the functional scans were spatially normalized using the unified segmentation approach to a standard template (Montreal Neurological Institute) and resampled to $3 \times 3 \times 3$ mm^3. This output file was then used to normalize the realigned functional data to standardize stereotactic space. Finally, the EPIs were smoothed using a Gaussian kernel of 8 mm full-width at half- maximum, detrended, and passed through a band-pass filter (0.01–0.08 Hz) to reduce low-frequency drift and high-frequency physiological noise. Nuisance covariates including head motion, white matter signals and cerebrospinal signals were regressed out from the timeseries. Regional time series

were extracted in each Human Brainnetome Atlas based brain regions by averaging the signals of all voxels within a region.

5. Transcriptome

5.1 Isolation and identification of exosome

Exosomes were isolated from plasma using the exoEasy Maxi Kit (Qiagen, Hilden, Germany) according to the standard protocol. The obtained exosomes were characterized by transmission electron microscopy (TEM) and ZetaView nanoparticle tracking analysis (NTA). For TEM imaging, exosome suspension was applied to an electron microscopy grid and fixed with 2% glutaraldehyde at room temperature. The samples were further stained with 2% uranyl acetate for 5 minutes, air-dried, and observed under a 120 kV transmission electron microscope. Particle size distribution and concentration were assessed by NTA performed on the ZetaView software (Particle Metrix GmbH) with the recommended settings.

5.2 Exosome RNA sequencing and data processing

Total RNA extracted from plasma exosomes was treated with DNase I (NEB, MA) for DNA removal. Strand-specific RNA sequencing libraries were prepared using the SMARTer Stranded Total RNA Kit (Clontech, Palo Alto, CA). The resulting libraries were sequenced on Illumina platform (San Diego, CA), and raw sequencing data were demultiplexed and converted to FASTQ format. Quality control, adapter trimming, and ribosomal RNA read removal were performed using BBDuk. The mRNAs sequence reads were aligned to the GRCh38 reference genome via HISAT2 program and

annotated based on the GENCODE database. Gene expression levels were quantified as mRNA counts and converted to transcripts per kilobase million (TPM) to enable cross-sample comparison.

Table S1. Anatomical and modified cyto-architectonic descriptions for brain regions

Label	Cyto-architectonic descriptions	Label	Cyto-architectonic descriptions
**Superior Frontal Gyrus**		**Superior Parietal Lobule**	
a1	*A8m, medial area 8*	m1	*A7r, rostral area 7*
a2	*A8dl, dorsolateral area 8*	m2	*A7c, caudal area 7*
a3	*A9l, lateral area 9*	m3	*A5l, lateral area 5*
a4	*A6dl, dorsolateral area 6*	m4	*A7pc, postcentral area 7*
a5	*A6m, medial area 6*	m5	*A7ip, intraparietal area 7 (hIP3)*
a6	*A9m, medial area 9*	**Inferior Parietal Lobule**	
a7	*A10m, medial area 10*	n1	*A39c, caudal area 39 (PGp)*
**Middle Frontal Gyrus**		n2	*A39rd, rostrodorsal area 39 (Hip3)*
b1	*A9/46d, dorsal area 9/46*	n3	*A40rd, rostrodorsal area 40 (PFt)*
b2	*IFJ, inferior frontal junction*	n4	*A40c, caudal area 40 (PFm)*
b3	*A46, area 46*	n5	*A39rv, rostroventral area 39 (PGa)*
b4	*A9/46v, ventral area 9/46*	n6	*A40rv, rostroventral area 40(PFop)*
b5	*A8vl, ventrolateral area 8*	**Precuneus**	
b6	*A6vl, ventrolateral area 6*	o1	*A7m, medial area 7 (PEp)*
b7	*A10l, lateral area10*	o2	*A5m, medial area 5 (PEm)*
**Inferior Frontal Gyrus**		o3	*dmPOS, dorsomedial parietooccipital sulcus (PEr)*
c1	*A44d, dorsal area 44*	o4	*A31, area 31 (Lc1)*
c2	*IFS, inferior frontal sulcus*	**Postcentral Gyrus**	
c3	*A45c, caudal area 45*	p1	*A1/2/3ulhf, area 1/2/3 (upper limb, head and face region)*
c4	*A45r, rostral area 45*	p2	*A1/2/3tonIa, area 1/2/3 (tongue and larynx region)*
c5	*A44op, opercular area 44*	p3	*A2, area 2*
c6	*A44v, ventral area 44*	p4	*A1/2/3tru, area1/2/3 (trunk region)*

Orbital Gyrus

d1	*A14m, medial area 14*
d2	*A12/47o, orbital area 12/47*
d3	*A11l, lateral area 11*
d4	*A11m, medial area 11*
d5	*A13, area 13*
d6	*A12/47l, lateral area 12/47*

Precentral Gyrus

e1	*A4hf, area 4(head and face region)*
e2	*A6cdl, caudal dorsolateral area 6*
e3	*A4ul, area 4(upper limb region)*
e4	*A4t, area 4(trunk region)*
e5	*A4tl, area 4(tongue and larynx region)*
e6	*A6cvl, caudal ventrolateral area 6*

Paracentral Lobule

f1	*A1/2/3ll, area1/2/3 (lower limb region)*
f2	*A4ll, area 4, (lower limb region)*

Superior Temporal Gyrus

g1	*A38m, medial area 38*
g2	*A41/42, area 41/42*
g3	*TE1.0 and TE1.2*
g4	*A22c, caudal area 22*
g5	*A38l, lateral area 38*
g6	*A22r, rostral area 22*

Middle Temporal Gyrus

Insular Gyrus

q1	*G, hypergranular insula*
q2	*vIa, ventral agranular insula*
q3	*dIa, dorsal agranular insula*
q4	*vId/vIg, ventral dysgranular and granular insula*
q5	*dIg, dorsal granular insula*
q6	*dId, dorsal dysgranular insula*

Precentral Gyrus

r1	*A23d, dorsal area 23*
r2	*A24rv, rostroventral area 24*
r3	*A32p, pregenual area 32*
r4	*A23v, ventral area 23*
r5	*A24cd, caudodorsal area 24*
r6	*A23c, caudal area 23*
r7	*A32sg, subgenual area 32*

MedioVentral Occipital Cortex

s1	*cLinG, caudal lingual gyrus*
s2	*rCunG, rostral cuneus gyrus*
s3	*cCunG, caudal cuneus gyrus*
s4	*rLinG, rostral lingual gyrus*
s5	*vmPOS, ventromedial parietooccipital sulcus*

lateral Occipital Cortex

t1	*mOccG, middle occipital gyrus*
t2	*V5/MT+, area V5/MT+*
t3	*OPC, occipital polar cortex*

h1 *A21c, caudal area 21*

h2 *A21r, rostral area 21*

h3 *A37dl, dorsolateral area37*

h4 *aSTS, anterior superior temporal sulcus*

Inferior Temporal Gyrus

i1 *A20iv, intermediate ventral area 20*

i2 *A37elv, extreme lateroventral area37*

i3 *A20r, rostral area 20*

i4 *A20il, intermediate lateral area 20*

i5 *A37vl, ventrolateral area 37*

i6 *A20cl, caudolateral of area 20*

i7 *A20cv, caudoventral of area 20*

Fusiform Gyrus

j1 *A20rv, rostroventral area 20*

j2 *A37mv, medioventral area37*

j3 *A37lv, lateroventral area37*

Parahippocampal Gyrus

k1 *A35/36r, rostral area 35/36*

k2 *A35/36c, caudal area 35/36*

k3 *TL, area TL (lateral PPHC, posterior parahippocampal gyrus)*

k4 *A28/34, area 28/34 (EC, entorhinal cortex)*

k5 *TI, area TI(temporal agranular insular cortex)*

t4 *iOccG, inferior occipital gyrus*

t5 *msOccG, medial superior occipital gyrus*

t6 *lsOccG, lateral superior occipital gyrus*

Amygdala

u1 *mAmyg, medial amygdala*

u2 *lAmyg, lateral amygdala*

Hippocampus

v1 *rHipp, rostral hippocampus*

v2 *cHipp, caudal hippocampus*

Basal Ganglia

w1 *vCa, ventral caudate*

w2 *GP, globus pallidus*

w3 *NAC, nucleus accumbens*

w4 *vmPu, ventromedial putamen*

w5 *dCa, dorsal caudate*

w6 *dlPu, dorsolateral putamen*

Thalamus

x1 *mPFtha, medial pre-frontal thalamus*

x2 *mPMtha, pre-motor thalamus*

x3 *Stha, sensory thalamus*

x4 *rTtha, rostral temporal thalamus*

x5 *PPtha, posterior parietal thalamus*

K6	*TH, area TH (medial PPHC)*	x6	*Otha, occipital thalamus*
**posterior Superior Temporal Sulcus**		x7	*cTtha, caudal temporal thalamus*
l1	*rpSTS, rostroposterior superior temporal sulcus*	x8	*lPFtha, lateral pre-frontal thalamus*
l2	*cpSTS, caudoposterior superior temporal sulcus*		

Table S2. Descriptive statistics of exposure conditions during the exposure sessions

**Variables**	**Heat**	**Control**
Temperature (°C)	32.1±0.5	21.8±0.3
Relative humidity (%)	51.1±1.8	50.7±1.4
$PM_{2.5}$ (μg/m^3)	3.8±2.0	5.5±2.1
NO_2 (ppb)	26.4±0.4	28.6±0.8
O_3 (ppb)	29.1±0.8	30.3±2.7
Skin temperature (°C)	29.5±1.2	33.6±0.6

Data are presented as mean ± standard deviation.

Table S3. Descriptive statistics of ambient exposure conditions during the three-day period preceding exposure

**Variables**	**Heat**	**Control**
Temperature (°C)	21.8±0.7	21.6±0.8
Relative humidity (%)	62.6±9.4	62.5±8.0
$PM_{2.5}$ (μg/m^3)	24.9±12.1	27.5±12.2
NO_2 (ppb)	27.4±0.1	27.5±21.2
O_3 (ppb)	29.1±2.2	29.5±2.2

Data are presented as mean ± standard deviation.

Table S4. Relative changes from baseline to post-exposure in psychological distress score and neurobehavioral indicators for the heat-exposure to the control-exposure sessions

Variable	Heat exposure (mean ± SD)		Control (mean ± SD)	
	1-h exposure	2-h exposure	1-h exposure	2-h exposure
**Psychological distress score**	2.5 (3.3)	4.1 (5.5)	0.3 (2.0)	0.0 (2.2)
**Schulte grid test**				
Responding time (s)				
Average time	0.8 ± 10.3	0.1 ± 10.1	-4.8 ± 6.9	-3.6 ± 8.2
Shortest time	74.0 ± 397.1	83.5 ± 421.9	-6.2 ± 8.8	-8.4 ± 9.3
**Stroop test**				
Responding time (ms)				
All	2.3 ± 4.7	1.4 ± 3.9	0.3 ± 4.7	-1.4 ± 4.2
Congruent	1.1 ± 4.7	0.2 ± 4.8	-0.4 ± 4.6	-2.2 ± 4.8
Incongruent	3.1 ± 5.1	2.3 ± 4.5	0.4 ± 5.4	-1.0 ± 4.6
Neutral	2.3 ± 6.5	1.1 ± 4.7	1.5 ± 5.9	-0.8 ± 5.6
Accuracy (%)				
All	-0.7 ± 2.4	-1.1 ± 2.0	-0.2 ± 1.7	-0.9 ± 1.9

Congruent	-0.5 ± 2.6	-1.3 ± 2.4	-1.4 ± 2.6	-1.1 ± 2.8
Incongruent	-0.9 ± 3.8	-1.0 ± 3.9	0.5 ± 3.6	-0.8 ± 3.6
Neutral	-0.2 ± 4.5	-0.9 ± 4.4	0.4 ± 4.6	-0.3 ± 5.3

Note: SD, standard deviation. **P-value* <0.05.

Table S5. Sex effects and interaction effects on psychological distress score and neurobehavioral indicators

Variable	Sex			Sex × intervention interaction		
	**β**	**SE**	***P*-value**	**β**	**SE**	***P*-value**
**1-h exposure**						
**Psychological distress score**	-0.11	0.81	0.89	-1.39	1.35	0.31
**Schulte grid test**						
Responding time (s)						
Average time	0.14	2.78	0.96	-1.80	4.68	0.70
Shortest time	-94.63	89.50	0.30	-136.26	146.17	0.36
**Stroop test**						
Responding time (ms)						
All	-1.34	1.59	0.41	1.78	2.32	0.45
Congruent	-1.52	1.50	0.32	1.06	2.37	0.66
Incongruent	-1.26	1.80	0.49	2.05	2.42	0.40
Neutral	-1.18	1.97	0.55	2.49	3.29	0.45
Accuracy (%)						
All	-0.59	0.66	0.38	1.26	1.10	0.26

Congruent	-0.93	0.83	0.27	1.65	1.37	0.24
Incongruent	-0.65	1.17	0.58	0.48	1.98	0.81
Neutral	0.17	1.46	0.91	3.00	2.26	0.19
**2-h exposure**						
**Psychological distress score**	0.48	1.15	0.68	-0.92	1.93	0.64
**Schulte grid test**						
Responding time (s)						
Average time	0.83	2.95	0.78	-2.35	4.94	0.64
Shortest time	-101.94	94.58	0.29	-150.11	156.79	0.35
**Stroop test**						
Responding time (ms)						
All	-0.52	1.23	0.67	0.18	2.08	0.93
Congruent	-0.74	1.47	0.62	-0.43	2.47	0.86
Incongruent	-0.48	1.39	0.73	0.03	2.34	0.99
Neutral	-0.32	1.61	0.84	1.91	2.71	0.48
Accuracy (%)						
All	-0.76	0.61	0.22	0.99	1.01	0.33

Congruent	-0.68	0.86	0.43	0.14	1.30	0.92
Incongruent	-0.94	1.18	0.43	1.37	1.98	0.49
Neutral	-0.55	1.64	0.74	1.48	2.36	0.53

Note: β, coefficient; SE, standard error.

Table S6. Brain regions differentially involved in response to heat exposure and the corresponding percentage changes in fMRI metrics

Brain regions	Hemisphere	MNI Coordinates (X, Y, Z)	Percentage changes (%, 95% CIs)		
			fALFF	DC	ReHo
**Inferior Frontal Gyrus**					
A44d, dorsal area 44	L	-46, 13, 24	-24.9 (-51.1, 1.3)	-47.0 (-87.5, -6.4)*	-23.9 (-55.6, 7.7)
	R	45, 16, 25	-30.4 (-49.8, -10.9)*	-28.4 (-68.4, 11.5)	-26.4 (-57.0, 4.2)
IFS, inferior frontal sulcus	L	-47, 32, 14	-25.6 (-46.3, -4.8)	-35.4 (-64.6, -6.1)*	-18.4 (-40.7, 3.9)
	R	48, 35, 13	-23.3 (-46.9, 0.3)	-38.3 (-67.9, -8.6)*	-15.6 (-41.9, 10.8)
A45r, rostral area 45	L	-49, 36, -3	-6.3 (-82.0, 69.4)	-79.6 (-141.6, -17.7)*	10.9 (-33.9, 55.8)
	R	51, 36, -1	4.3 (-172.4, 181.0)	-62.1 (-177.0, 52.9)	100.1 (-88.5, 288.6)
**Precentral Gyrus**					
A4tl, area 4	L	-52, 0, 8	-60.9 (-95.7, -26.1)*	-33.0 (-86.7, 20.7)	-42.6 (-102.0, 16.7)
	R	54, 4, 9	-37.4 (-66.4, -8.5)*	-15.1 (-72.8, 42.6)	-19.6 (-70.5, 31.3)
A6cvl, caudal ventrolateral area 6	L	-49, 5, 30	-16.0 (-51.1, 19.0)	-29.8 (-84.2, 24.7)	-12.2 (-70.1, 45.7)
	R	51, 7, 30	-26.8 (-48.2, -5.3)*	-35.2 (-76.5, 6.0)	-40.8 (-84.6, 2.9)
**Lateral Occipital Cortex**					

mOccG, middle	L	-31, -89, 11	34.0 (9.5, 58.6)	82.4 (-32.8, 197.8)	20.0 (-14.9, 54.8)
occipital gyrus	R	34, -86, 11	31.0 (7.2, 54.9)	113.1 (0.3, 226.0)	53.6 (18.8, 88.3)*
V5/MT+, area V5/MT+	L	-46, -74, 3	10.4 (-6.1, 27.0)	13.8 (-22.5, 50.0)	14.7 (-3.7, 33.1)
	R	48, -70, -1	21.2 (1.1, 41.3)	36.4 (2.7, 70.0)	32.7 (12.1, 53.3)*
**Hippocampus**					
rHipp, rostral	L	-22, -14, -19	-7.7 (-18.7, 3.4)	-17.3 (-37.0, 2.4)	-18.8 (-28.7, -8.8)*
hippocampus	R	22, -12, -20	-6.3 (-19.4, 6.9)	-11.2 (-34.4, 12.0)	-3.9 (-15.2, 7.4)

Note: CI, confidence interval; fMRI, functional magnetic resonance imaging; fALFF, fractional amplitude of low frequency fluctuation; DC, degree centrality; ReHo, regional homogeneity. L and R denote left and right brain hemispheres, respectively. **FDR-value* <0.1.

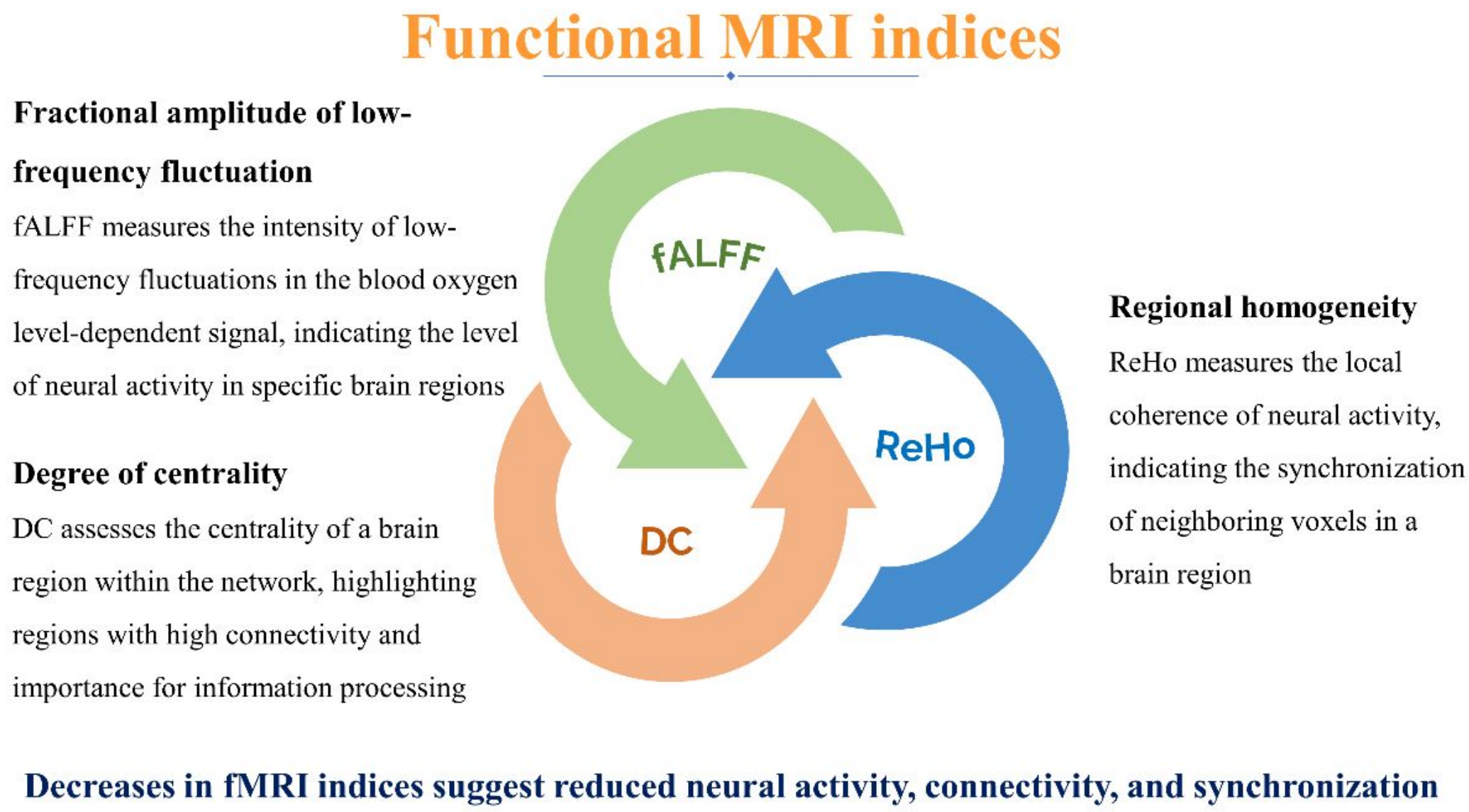

Figure S1. Overview of functional MRI indices.

MRI, magnetic resonance imaging; fALFF, fractional amplitude of low frequency fluctuation; DC, degree centrality; ReHo, regional homogeneity.

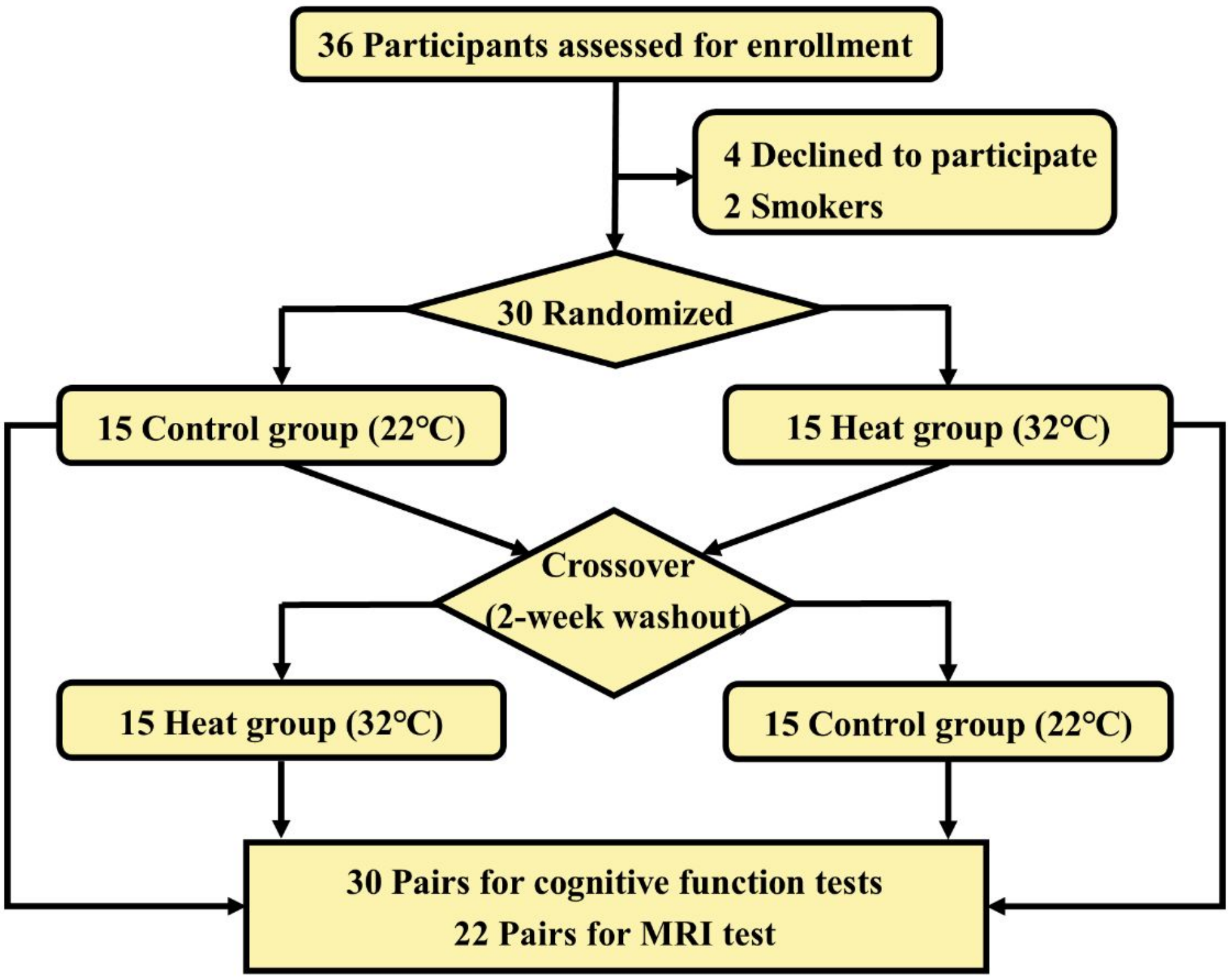

Figure S2. Flowchart of participation in the present study.

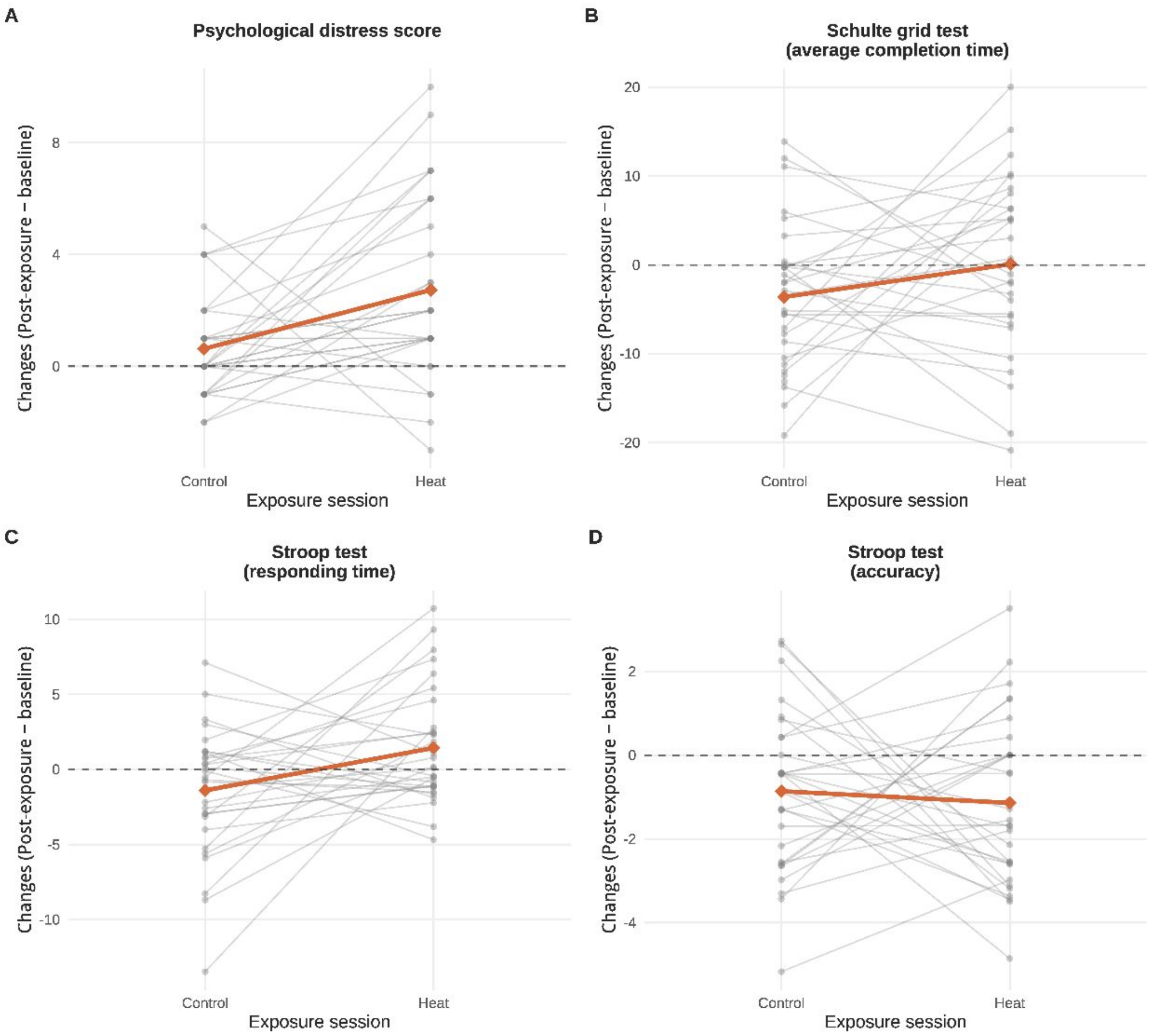

Figure S3. Individual response patterns across psychological and cognitive measures.

Individual trajectories (gray lines) and means (red diamonds) for (A) psychological distress score, (B) average completion time of Schulte grid test, (C) responding time of Stroop test, and (D) accuracy of Stroop test. Each panel shows within-subject changes from baseline to post-exposure under control- and heat-exposure sessions.

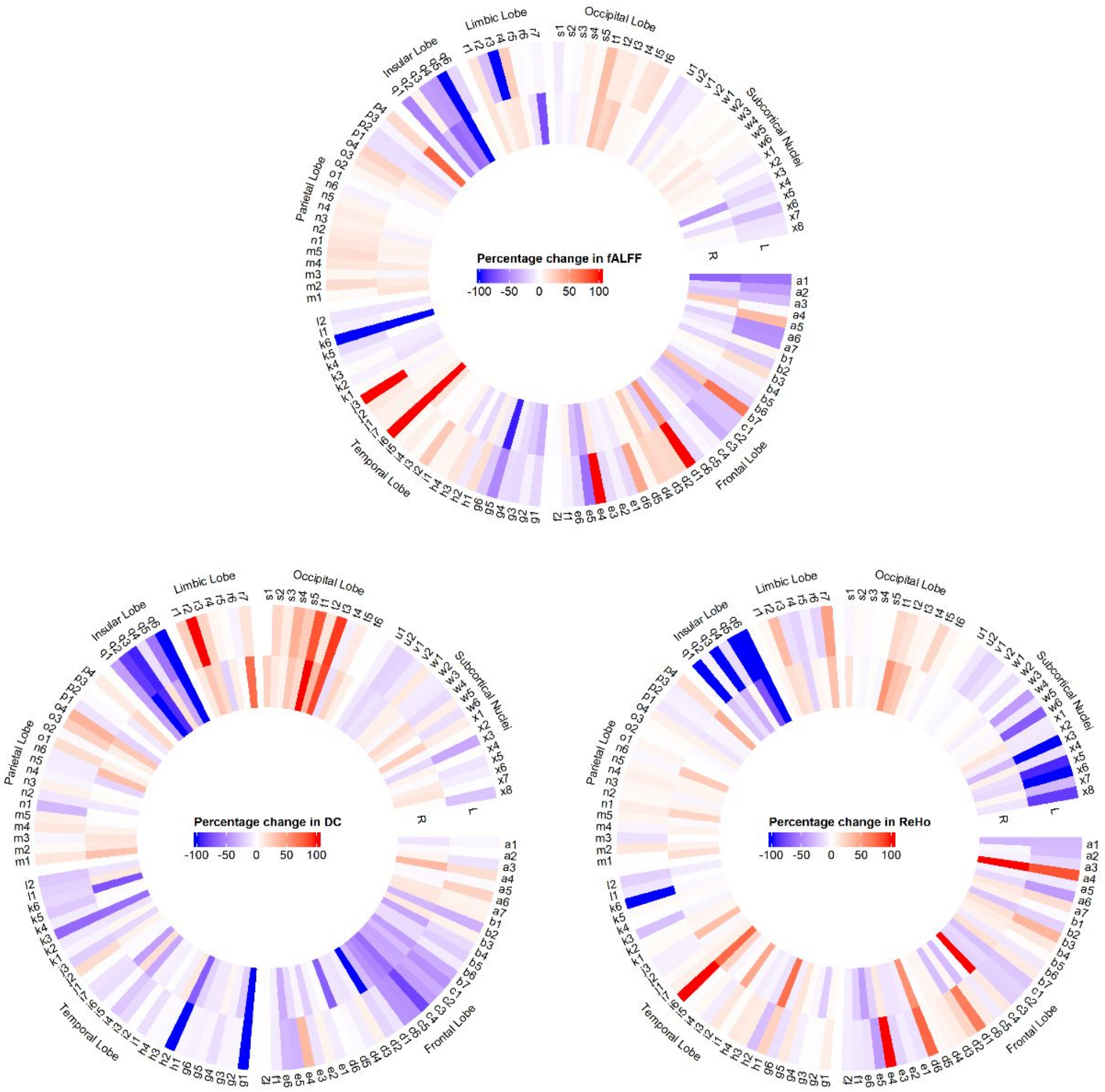

Figure S4. Percentage changes in fMRI metrics comparing the heat-exposure to the control-exposure sessions.

fMRI, functional magnetic resonance imaging; fALFF, fractional amplitude of low frequency fluctuation; DC, degree centrality; ReHo, regional homogeneity.

Figures were mapped by brain connectome atlas with 123 segments separated in two layers representing left and right brain hemispheres. L and R denote the left and right brain hemispheres, respectively. Anatomical and modified cyto-architectonic descriptions for brain regions was shown in Table S1.

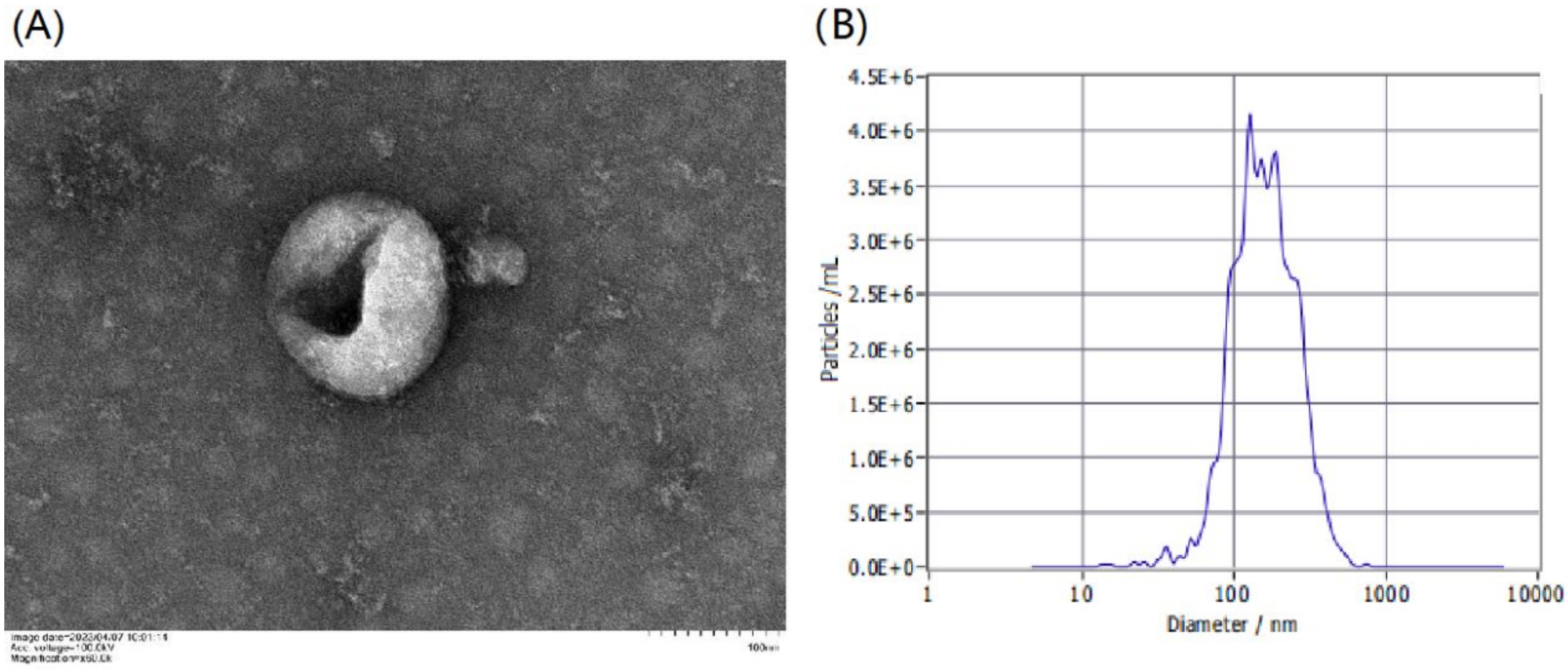

Figure S5. Exosome examination and validation.

(A) Representative images of isolated exosomes; (B) Size distribution measurement of isolated exosomes.

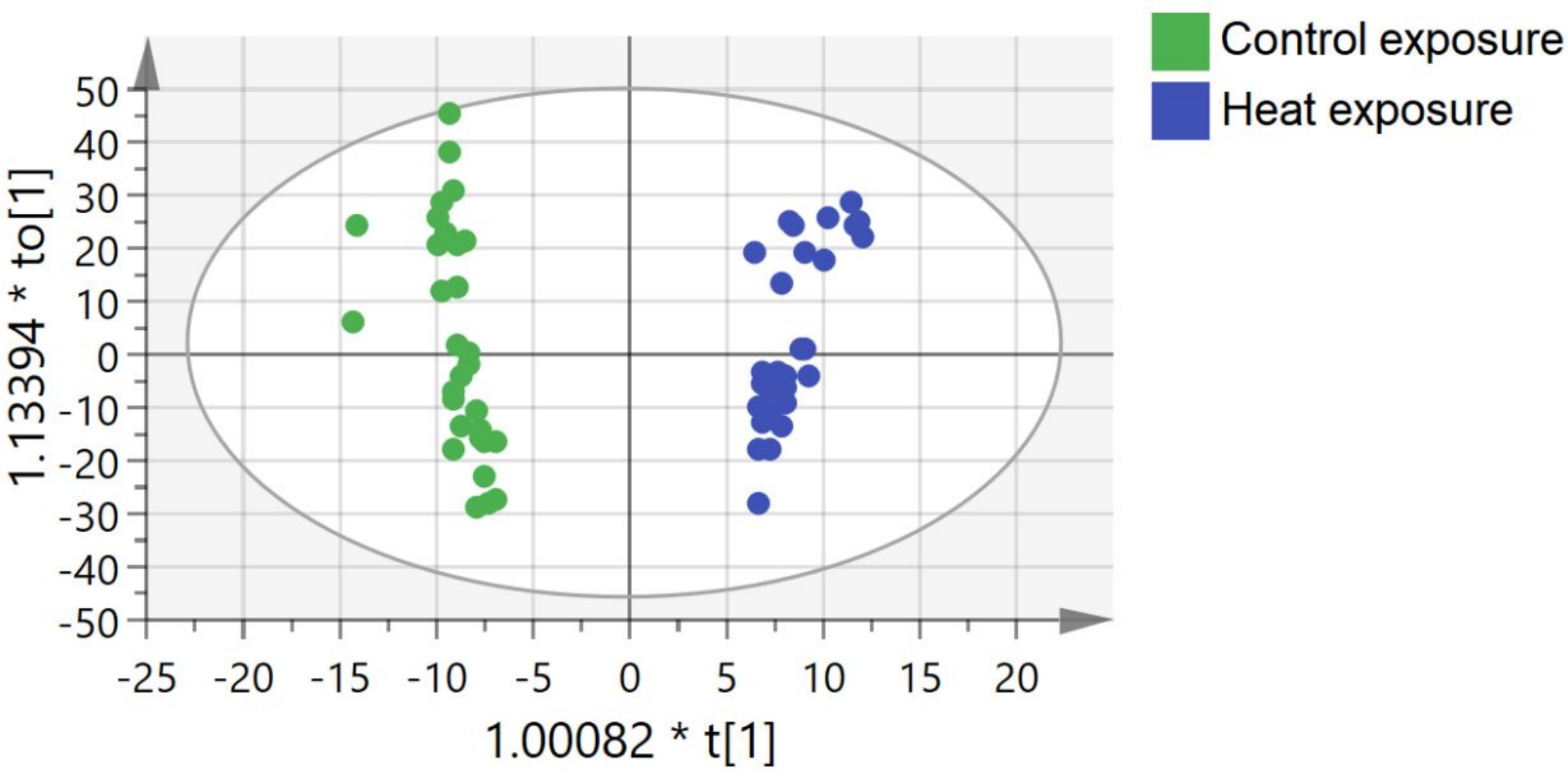

Figure S6. Score plot from the OPLS-DA analysis for the transcriptomic analysis.

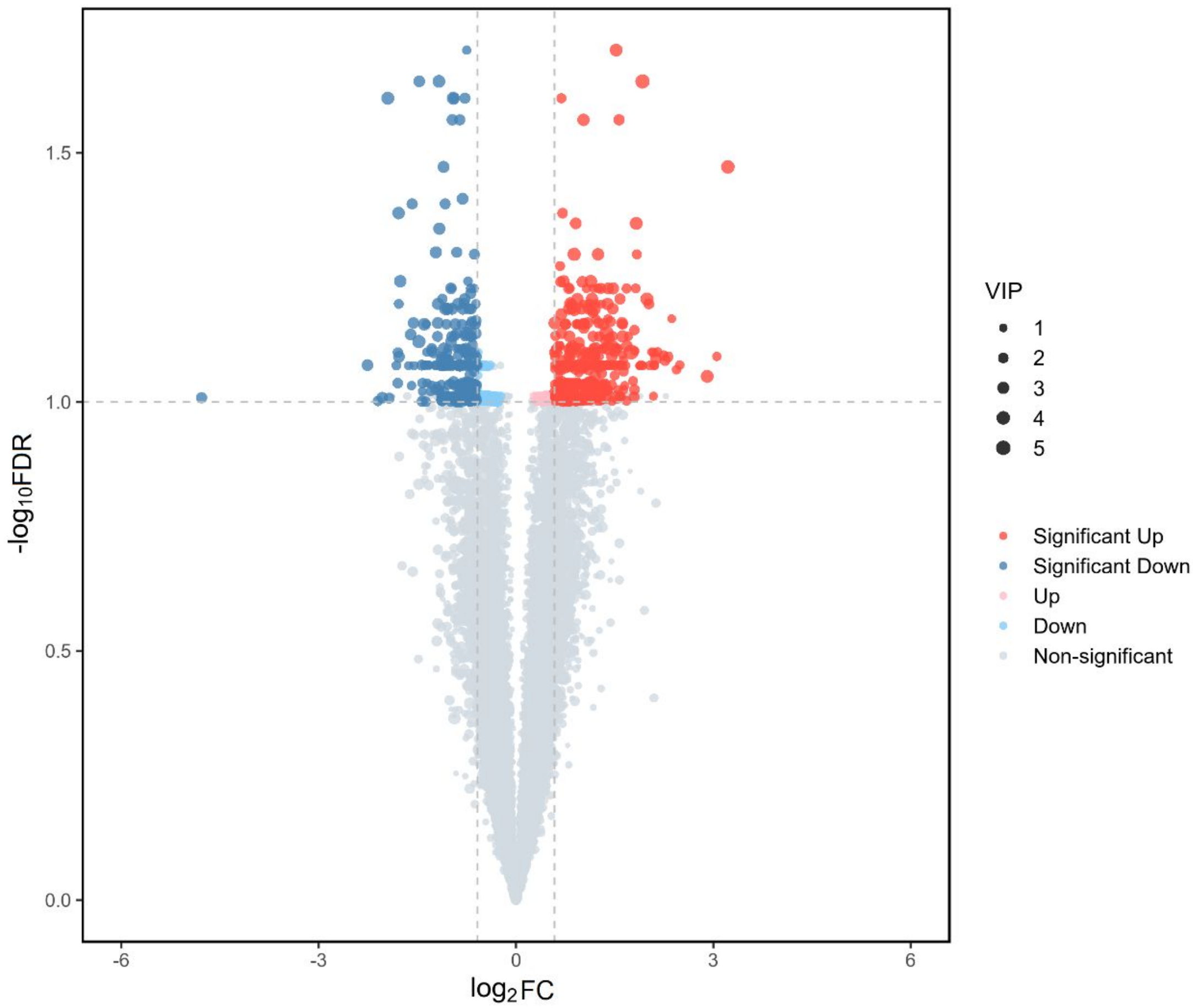

Figure S7. Volcano plot for the transcriptomic analysis.

VIP, variable importance in the projection; FDR, false discovery rate; FC, fold change. VIP > 1, FC > 1.5, and FDR <0.1 were set as the thresholds for identifying differentially expressed genes. Red dots and blue dots represent the upregulated and downregulated genes, respectively.
